# Prospective Upfront Next-Generation Sequencing for Advanced Non-Small Cell Lung Cancer: Real-World Outcomes from the Ion Chiricuță Oncology Institute

**DOI:** 10.3390/ijms26073403

**Published:** 2025-04-05

**Authors:** Alexandra Cristina Preda, Nicolae Todor, Bogdan Cârlan, Adelina-Dadiana Kubelac-Varro, Dana Ioana Iancu, Cristina Mocan, Mariana Bandi Vasilica, Milan-Paul Kubelac, Cătălin Vlad, Tudor Eliade Ciuleanu

**Affiliations:** 1 Oncology Institute “Prof. Dr. Ion Chiricuță” 34–36 Republicii Street, 400015 Cluj-Napoca, Romania; 2 Faculty of Medicine, “Iuliu Hațieganu” University of Medicine and Pharmacy, 8 Victor Babeș Street, 400012 Cluj-Napoca, Romania; 3 Medlife Oncology Hospital, 65A Carierei Street, 500062 Brașov, Romania; 4 STAR Institute, Babeș-Bolyai University, 1 Mihail Kogălniceanu Street, 400347 Cluj-Napoca, Romania

**Keywords:** next-generation sequencing, FoundationOne, non-small cell lung cancer, personalized treatment

## Abstract

Upfront Next-Generation Sequencing (NGS) is increasingly recommended in advanced NSCLC to guide targeted therapy. This prospective single-center study in Romania evaluated routine, upfront NGS in advanced NSCLC at baseline (tissue and/or liquid) and progression (liquid). Baseline FoundationOne NGS (tissue/liquid) was performed in 119 consecutive stage IV NSCLC patients, along with PD-L1 immunohistochemistry (IHC, SP263). Liquid biopsy was repeated at progression. Turnaround time (TAT), the prevalence of actionable targets, and clinical utility were assessed. Patients were predominantly male (68.1%) with a median age of 62 years (range 30–86). Most had ECOG PS 0–1 (79%) and non-squamous histology (67.2%). Never-smokers accounted for 25.2%. The median TAT for the NGS results was 9 days (range 5–21). Overall, 671 genetic alterations were detected in 149 genes. The mean number of distinct mutations per patient dropped from 5.6 at baseline to 4.3 at progression. Tissue samples yielded more alterations (6 per patient) than baseline liquid biopsies (4.6). Squamous tumors had more alterations (7.1 vs. 4.8 in non-squamous), and the number of smokers exceeded that of never-smokers (6 vs. 4.5). TP53 was the most frequent (70.59%). Actionable variants were found in 74.8% of patients, though only 35.3% received personalized therapy, largely due to performance status deterioration, reimbursement, or trial availability barriers. Common targets in non-squamous tumors included EGFR (21%), KRAS G12C (11%), NF1 (11%), and ERBB2 (6%); in squamous tumors, common targets included NF1 (24%), PIK3CA (18%), and ERBB2 (8%). Among smokers, driver mutations were often NF1 (15%), PIK3CA (11%), KRAS G12C (9%), and ERBB2 (8%); never-smokers were dominated by EGFR (45%), NF1 (15%), and KRAS G12C (8%). TMB ≥ 10 mut/Mb was seen in 26.9%; no patients were MSI-H. PD-L1 TPS was <1% in 33% of patients, 1–49% in 20%, ≥50% in 18%, and unknown in 29%. Upfront NGS offers rapid, comprehensive genomic data, guiding tailored therapies and trials in advanced NSCLC. Liquid rebiopsy at progression further refines treatment decisions.

## 1. Introduction

According to GLOBOCAN 2022, lung cancer is the most diagnosed malignancy in men (1.57 million new cases) and the second in women (0.91 million), remaining the top cause of cancer mortality worldwide (1.23 million deaths) [1]. In Romania, there were 11,716 new cases of lung cancer reported in 2022, ranking third after colorectal and breast cancer and accounting for 11.2% of all new cancer cases. In the same year, lung cancer resulted in 10,530 deaths, making it the leading cause of cancer-related mortality at 18.7%. The five-year prevalence stands at 15,500 cases, equivalent to 81.4 per 100,000 inhabitants [1]. Treatment decisions require a multidisciplinary team, considering histology, stage, performance status, comorbidities, and molecular genomic testing. Given the rise in FDA/EMA-approved molecular targets and clinical trials, NCCN, ASCO, and ESMO now recommend comprehensive genomic analysis at baseline and progression.

Although NGS testing was historically limited in Romania, it became available in 2024 through a National Program. This prospective pilot study (April 2022–September 2024) introduced NGS analysis in our Institute, estimating the prevalence of actionable alterations found via FoundationOne at baseline (tumor/liquid) and progression (liquid) and assessing how these findings influenced treatment choices (targeted therapies or trial enrollment).

## 2. Results

### 2.1. Patients and NGS Testing

Between April 2022 and September 2024, 122 patients were screened. However, 1 (0.8%) was excluded due to a concomitant melanoma, and 2 could not undergo F1CDx or liquid biopsy, leaving 119 for analysis. The median turnaround time for FoundationOne and PD-L1 testing was nine days (range 5–21). Among the 119 patients, 80 (67.2%) had non-squamous histology, and 39 (32.8%) had squamous histology. The median age was 62, and there were 81 (68.1%) males. A total of 94 (79%) had ECOG PS 0–1, and 30 (25.2%) were never-smokers (Table 1). Of the 119 patients, 43 (36.1%) had tissue testing at baseline, 99 (83.2%) had a baseline liquid biopsy, and 34 (28.6%) had liquid biopsy at progression. In the last category, 11 patients (9.2%) that had an initial single-gene assay were tested according to the second inclusion criterion, with NGS testing conducted only at progression.

### 2.2. Overall Genetic Abnormalities (F1CDx Tissue at Baseline, F1LCDx Blood at Baseline, F1LCDx Blood at Progression)

Of 119 evaluable patients, 671 total genetic abnormalities were identified in 149 distinct genes (45.9% of the 324 tested genes). On average, 5.6 alterations per patient (range: 0–16) were found (see Table 2 and Table S1). Four patients had no detectable mutations.

### 2.3. Genetic Abnormalities Found at Baseline (Tissue, Blood)

Of the 119 patients with valid results, 104 (87.4%) underwent NGS (tissue, blood, or both) at baseline. In total, 587 genetic abnormalities were identified across these 104 patients, with an average of 5.6 per patient (range, 0–14). Among the 324 genes analyzed, 139 distinct genes (42.9%) carried alterations, while 3 patients had no detectable mutations (Table 2 and Table S2). The most frequent changes, detected in >10% of patients, involved TP53, KRAS, CDKN2A/B, STK11, EGFR, NF1, and KEAP1. Variants in DNMT3A, TET2, and ASXL1 may represent clonal hematopoiesis.

### 2.4. Genetic Abnormalities at Baseline in Tissue Biopsies Only

Only 43 of the 119 patients (36.1%) had sufficient tumor tissue for baseline NGS, as many opted for liquid biopsy instead of repeating a tissue biopsy. This can represent a limitation of this study. Among these 43 individuals, 257 genetic abnormalities were identified, with an average of 6 per patient (range, 1–13). Overall, 98 genes (30.2% of the 324 tested) were mutated (Table 2 and Table S3). Genes found to be mutated in >10% of the patients at baseline included TP53, CDKN2A/B, MTAP, EGFR, KRAS, STK11, NF1, PIK3CA, PRKCI, PTEN, KEAP1, SOX2, and TERC.

### 2.5. Genetic Abnormalities at Baseline in Liquid Biopsies Only

Of the 119 patients, 99 (83.2%) had a valid baseline liquid biopsy for NGS. In these 99 individuals, 453 genetic abnormalities were identified, with an average of 4.6 per patient (range, 0–11). In total, 113 distinct genes (34.9% of the 324 tested) harbored mutations (Table 2 and Table S4). The most frequently mutated genes (>10% of patients) included TP53, KRAS, STK11, EGFR, and NF1, while changes in DNMT3A, TET2, and ASXL1—also found in >10% of patients—may reflect clonal hematopoiesis.

### 2.6. Genetic Abnormalities Found at Progression in Liquid Biopsy

Of the 119 patients, 34 (28.6%) experienced disease progression and had valid blood samples for NGS at that time, yielding 146 genetic abnormalities. One patient had no detectable mutation. The mean number of alterations per patient was 4.3 (range, 0–10). In total, 52 distinct genes (16% of the 324 tested) harbored abnormalities (Table 2 and Table S5). The most frequently mutated genes (>10% of patients) included TP53, KRAS, STK11, KEAP1, NF1, and CDKN2A. Notably, DNMT3A, ASXL1, ATM, CHEK2, and TET2 were also altered in >10% of patients, potentially representing clonal hematopoiesis.

### 2.7. Mutation Frequency: Diagnosis vs. Progression

**Figure 1:**
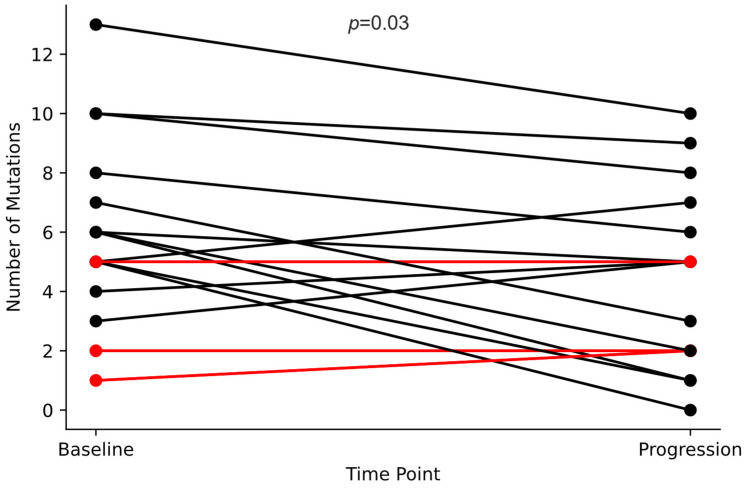
Trend in mutation count for patients tested at both baseline and progression (*n* = 19). Red lines represent two patients.

Among the 104 patients who underwent FoundationOne testing at baseline (F1CDx Tissue and/or F1LCDx Blood) and the 34 who underwent FoundationOne testing at progression (F1LCDx Blood), the mean mutation count was 5.6 and 4.3 per patient, respectively (*p* = 0.03, Student’s *t*-test). Among the 19 patients tested at both baseline and progression, the average mutation count decreased from 5.5 to 4.2 per patient (*p* = 0.03), as detailed in Figure 1.

### 2.8. Genetic Abnormalities Found at Baseline (Tissue, Blood) by Histology

#### 2.8.1. Non-Squamous NSCLC

Of the 119 evaluable patients, 80 had non-squamous NSCLC, of whom 66 underwent baseline NGS (tissue, liquid, or both), revealing 316 genetic abnormalities. Two patients had no detectable mutations. The average mutation count per patient was 4.8 (range, 0–12). Overall, 100 distinct genes (30.8% of the 324 tested) were altered (Table 2 and Table S6).

**Figure 2:**
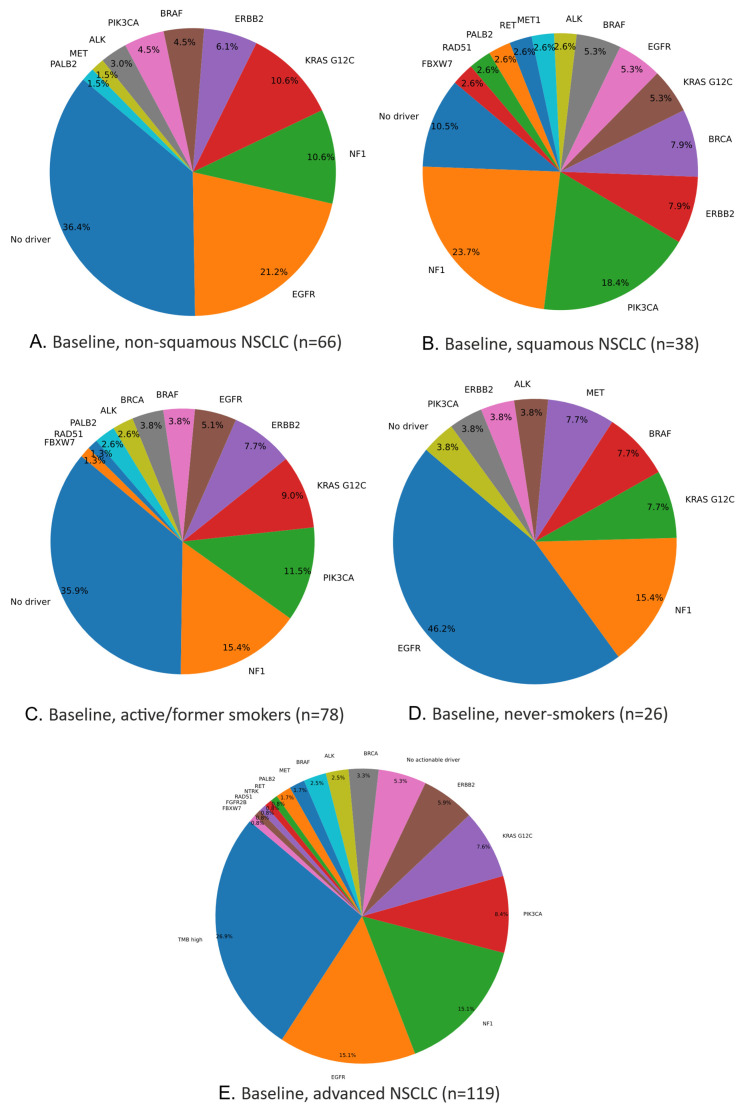
Actionable mutations in NSCLC at baseline, in non-squamous NSCLC (**A**), squamous NSCLC (**B**), active/former smokers (**C**), never-smokers (**D**), and overall (**E**).

Among these non-squamous cases, genes observed in >10% of patients included TP53, KRAS, STK11, EGFR, CDKN2A/B, KEAP1, and NF1, while changes in DNMT3A, TET2, ASXL1, and ATM—also observed in >10%—may indicate clonal hematopoiesis. Figure 2A illustrates actionable mutations: the most frequent driver was EGFR (21%), followed by NF1, KRAS G12C, ERBB2, BRAF, PIK3CA, ALK, MET, and PALB2. Notably, 36% of non-squamous NSCLC cases lacked any identified actionable driver.

#### 2.8.2. Squamous NSCLC

Among the 119 evaluable patients, 39 had squamous NSCLC, of whom 38 underwent baseline NGS (tissue, blood, or both), yielding 271 genetic abnormalities. One patient had no detectable mutation. The mean number of alterations per patient was 7.1 (range, 0–14). Overall, 94 distinct genes (29% of the 324 tested) harbored mutations (Table 2 and Table S7).

Genes present in >10% of these squamous cases included TP53, CDKN2A/B, NF1, PIK3CA, PTEN, PRKCI, KEAP1, MTAP, MYC, NOTCH1, SOX2, TERC, ARID1A, and KRAS. Variants in DNMT3A, ASXL1, TET2, and CHEK2 (also >10%) could indicate clonal hematopoiesis.

Figure 2B illustrates the actionable mutations and their frequencies. The most common actionable driver in this squamous subgroup was NF1 (23.68%), followed by PIK3CA, ERBB2, BRCA, KRAS G12C, EGFR, BRAF, ALK, MET, RET, PALB2, RAD51, and FBXW7. Notably, 10% of patients lacked any identified actionable driver.

### 2.9. Genetic Abnormalities Found at Baseline (Tissue, Blood) by Smoking Status

#### 2.9.1. Active or Past Smokers

Among the 119 patients, 89 (74.8%) were active or former smokers, of whom 78 (87.6%) underwent baseline NGS (tissue, liquid, or both). In these 78 patients, 471 genetic abnormalities were identified, with 1 patient having no detectable mutation. The mean mutation count was six (range, 0–14). Overall, 127 distinct genes—39.1% of the 324 tested—were altered (Table 2 and Table S8).

Genes observed in >10% of active/former smokers included TP53, KRAS, STK11, CDKN2A/B, NF1, KEAP1, PIK3CA, and RB1, while DNMT3A, ASXL1, TET2, ATM, and CHEK2—also observed in >10%—may indicate clonal hematopoiesis. Figure 2C shows the actionable mutations and their frequencies; the most common driver was NF1 (15%), followed by PIK3CA (11%), KRAS G12C, ERBB2, EGFR, BRAF, BRCA, ALK, PALB2, RAD51, and FBXW7. Together, NF1 and PIK3CA accounted for 26% of actionable events in this subgroup, while 36% of patients had no identifiable oncogenic driver.

#### 2.9.2. Never-Smokers

Among the 119 patients, 30 (25.2%) were never-smokers, of whom 26 (86.7%) underwent baseline NGS (tissue, liquid, or both), revealing 116 genetic abnormalities in total. Two patients had no detectable mutations. The mean mutation count was 4.5 (range, 0–10). Overall, 57 distinct genes (17.6% of the 324 tested) were altered (Table 2 and Table S9). Genes appearing in >10% of never-smokers included TP53, EGFR, CDKN2A/B, NF1, KRAS, MTAP, and SMARCA4, while variants in DNMT3A, TET2, and CBL—also observed in >10%—suggest possible clonal hematopoiesis. Figure 2D shows the actionable mutations and their frequencies; EGFR (45%) was the most prevalent driver, followed by NF1, KRAS G12C, BRAF, MET, ALK, ERBB2, and PIK3CA. Notably, only 4% of these never-smokers lacked an identifiable actionable driver.

### 2.10. Actionable Genetic Changes and Clinical Applications

Of 119 patients, 30 (25.2%) had no actionable alterations or genomic signatures, while 89 (74.8%) had at least one. TMB ≥ 10 was considered “actionable” due to the FDA’s tumor-agnostic checkpoint inhibitor approval. Overall, 68 patients (57.1%) had one actionable alteration, 18 (15.1%) had two, and 3 (2.5%) had three. Known lung cancer genes (EGFR, KRAS G12C, ALK, MET, RET) were found in 33 patients; 11 had genes relevant to both lung and other cancers (ERBB2, BRAF V600, NTRK), and 37 had genes typically linked to other malignancies (NF1, PIK3CA, PALB2, FGFR2B, FBX7, RAD51).

All 119 patients were assessed for MSI-H and TMB ≥10, with none being MSI-H and 32 (26.9%) being TMB-high. Among the 89 patients with actionable findings, 42 (47.2%)—35.3% of the entire cohort—received personalized/targeted therapy (Table 3). Of these patients, 18 received EGFR-TKIs (reimbursed—16 osimertinib, 2 afatinib), 7 received anti-KRAS G12C treatment (clinical trial—adagrasib), 3 received ALK-TKIs (reimbursed—2 alectinib, 1 brigatinib), 2 received anti-MET therapy (clinical trial—telisotuzumab vedotin), 1 received anti-RET therapy (clinical trial—pralsetinib), 1 received anti-FGFR2b therapy (clinical trial—bemarituzumab), 1 received anti-ERBB2 therapy (own source—trastuzumab deruxtecan), 1 received anti-PIK3CA therapy (own source—alpelisib), and 8 received immunotherapy for TMB-high (clinical trial—nivolumab plus ipilimumab). The remaining 47 (52.8%) did not receive personalized/targeted therapy due to poor performance status or rapid progression (16), a lack of reimbursement (13), the absence of EMA reimbursement or an adequate clinical trial available (10), alternate guideline indications (6), or the case where initial actionable mutations were no longer found at progression and a non-targeted treatment was considered more appropriate (2).

### 2.11. PD-L1 Expression at Baseline

Of the 119 patients, 87 underwent PD-L1 immunohistochemistry (Ventana SP263) at baseline, primarily limited by insufficient tissue in some cases; 2 tests failed technically. The incomplete characterization of patients’ PD-L1 status may limit this study’s results.

Consequently, 85 yielded valid PD-L1 results: 45.9% were PD-L1-negative (TPS < 1%), 28.2% had low/moderate expression (TPS 1–49%), and 25.9% had high expression (TPS ≥ 50%).

In routine clinical care, PD-L1 expression is crucial for selecting therapy in non-oncogene-driven NSCLC, as pembrolizumab, cemiplimab, or atezolizumab monotherapy is preferred first-line for high expressors (TPS ≥ 50%), whereas combination chemo-immunotherapy can be used regardless of PD-L1 status. In our cohort, 22 patients fell into the high-PD-L1 category, of whom 9 received monotherapy, and another 9 received combination immunotherapy. Two patients were treated with platinum doublets (due to immunotherapy contraindications), and two experienced rapid clinical deterioration, ultimately receiving only symptomatic care.

### 2.12. Particular Situations

#### 2.12.1. Clonal Hematopoiesis (CH)

Clonal hematopoiesis of indeterminate potential (CHIP) is an age-associated process in which hematopoietic stem cells acquire somatic mutations that drive clonal expansion. The comprehensive genomic profiling of solid tumors often identifies these non-tumor alterations, reflecting background clonal hematopoiesis rather than true tumor-derived mutations. Commonly implicated genes include ASXL1, ATM, CBL, CHEK2, DNMT3A, IDH2, JAK2, KMT2D (MLL2), MPL, MYD88, SF3B1, TET2, and U2AF1, although TP53, BRAF, KRAS, and NRAS can also occasionally be involved [2]. Distinguishing CHIP from tumor-specific mutations typically requires matched peripheral blood mononuclear cell sequencing and dedicated bioinformatic algorithms. When interpreting the FoundationOne results, any variants in known CH genes are flagged (e.g., printed in red), but clinical context remains essential [2].

#### 2.12.2. Genomic Signatures: MSI and TMB

All 119 patients in this study were microsatellite-stable (MSS) according to FoundationOne testing, with no instances of patients being microsatellite instability-high (MSI-H). In addition, we considered tumor mutational burden (TMB ≥ 10 mut/Mb) an “actionable” biomarker, in line with the FDA’s tissue-agnostic approval of checkpoint inhibitors for TMB-high tumors. Consequently, 32 patients (26.9%) were categorized as TMB-high, broadening potential immunotherapeutic options within this advanced NSCLC population.

## 3. Discussion

### 3.1. Upfront NGS (Tissue and Liquid) Gains Ground

This study is among the first Romanian series to prospectively apply FoundationOne NGS (tissue and/or liquid at baseline, liquid at progression). Previously, routine testing in Romania focused on EGFR and ALK, whereas comprehensive NGS broadens mutation detection, enabling on-label targeted therapies and referral to clinical trials (some of our patients were included in trials targeting EGFRins20, ERBB2, FGFR2B, KRAS G12C, MET, NF1, RET, ROS1, and TMB-high).

Of 119 patients, 74.8% had at least one actionable alteration, yet only 35.3% ultimately received targeted therapy. The main obstacle was rapid clinical deterioration (often ECOG PS 2), which precluded waiting for the NGS results; other barriers included a lack of reimbursement, no suitable trials, or off-label restrictions—challenges also reported elsewhere [3].

Compared to single-gene testing (SGT), NGS also reduces overall turnaround time and the need for repeat biopsies. Studies from the United States and Spain report faster results with NGS (2.0 vs. 4.7–4.8 weeks and 10 vs. 15.48 days, respectively) [4,5]. In our series, FoundationOne plus PD-L1 testing results were returned in a median of 9 days (range, 5–21), aligning with prior data (8.8 days) [6].

Liquid biopsy offers a non-invasive option repeatable at progression, although it may miss low-frequency mutations and can be confounded by clonal hematopoiesis [2]. In our series, clonal-hematopoiesis-associated genes were more common in plasma (25.1% vs. 3.9% in tissue), and we observed a median of five alterations per patient overall.

Professional societies (ASCO, ESMO, NCCN) increasingly endorse the upfront testing of multiple molecular markers, making comprehensive NGS plus PD-L1 IHC more cost-effective and clinically efficient than sequential testing [4,7,8]. Nonetheless, debates persist over universal NGS use vs. a more selective approach or waiting until progression [3,9,10]. Some studies link upfront NGS to improved survival [11,12], while others find no difference when resources are limited [13], yet ESMO generally favors multiplex (NGS) testing [14].

### 3.2. Frequent Non-Druggable Genes

TP53 was the most commonly mutated (70.59%), particularly in squamous tumors (81.58%) and among current/former smokers, correlating with worse outcomes and resistance [15,16,17,18]. Other tumor suppressor genes (KEAP1, STK11), which can impair immunotherapy responses in KRAS-mutant cancers [19,20,21,22], showed slightly increased frequency from baseline to progression.

### 3.3. Actionable Alterations and Shifting Frequencies

Table 4 shows the prevalence of actionable genetic alterations at baseline (tissue and/or liquid) and at progression (liquid). Most variants (e.g., BRAF, BRCA2, EGFR, ERBB2, KRAS, NF1, PIK3CA) appeared at both time points, with some detected only at baseline (RET in tissue; ALK, BRCA1 in liquid) or only at progression (FGFR2B, NTRK). Three frequency trends emerged between baseline and progression: (1) increases (e.g., KRAS), (2) decreases (e.g., EGFR, ERBB2, PIK3CA), and (3) relative stability (e.g., NF1, BRCA, BRAF).

KRAS—one of the most frequent oncogenes—nearly doubled in frequency from baseline (18.6% tissue; 22.2% liquid) to progression (35.3% liquid). Within these KRAS mutations, the G12C variant (responsible for ~40% of KRAS mutations in lung cancer) made up ~28.1% [23]. As expected, KRAS mutations were more common in non-squamous vs. squamous NSCLC (33.5% vs. 10.6%) and in current/former vs. never-smokers (29.5% vs. 11.5%) [24].

Meanwhile, EGFR, ERBB2, and PIK3CA decreased two- to five-fold from baseline to progression; for example, EGFR fell from 18.6% (tissue) and 13.1% (liquid) to 8.8%. This likely reflects effective targeted therapy, as “clearing” EGFR in plasma can signify TKI response [25]. A new liquid biopsy at progression can identify on-target resistance mutations (e.g., T790M, C797S) or off-target drivers (MET, RET, KRAS, BRAF, PIK3CA amplifications/rearrangements), prompting therapy revisions [26]. However, liquid biopsy cannot detect histologic transformations (e.g., small-cell), necessitating tissue rebiopsy when indicated [26].

### 3.4. Comparison with Cancer Genome Atlas Results, Ten Years On

A decade ago, The Cancer Genome Atlas (TCGA) reported high mutation rates and significantly mutated genes in both lung adenocarcinomas and squamous cell carcinomas [15,27]. In 230 resected, untreated adenocarcinomas (non-squamous NSCLC), the mean mutation rate was 8.9 mutations/Mb, with 18 genes significantly mutated. TP53 appeared in 46% (less often than in squamous NSCLC), while KRAS (33%) and EGFR (14%) were mutually exclusive. Other recurrent genes included BRAF (10%), PIK3CA (7%), MET (7%), and RIT1 (2%), plus tumor suppressors STK11 (17%), KEAP1 (17%), NF1 (11%), RB1 (4%), and CDKN2A (4%); chromatin modifiers SETD2 (9%), ARID1A (7%), and SMARCA4 (6%); splicing genes RBM10 (8%) and U2AF1 (3%); and the MYC pathway regulator MGA (8%), which was often mutually exclusive with focal MYC amplification [27].

In our 66 non-squamous patients with FoundationOne data, all but MGA and RIT1 (not on the panel) were found in >1.5% of cases at baseline. We also observed MTAP, BCOR, ERBB2, APC, KDM6A, and others in >1.5%, while DNMT3A, TET2, ASXL1, ATM, JAK2, SF3B1, CBL, KMT2D (MLL2), and CHEK2 suggested clonal hematopoiesis, for a total of 100 distinct mutated genes (Table 5 and Table S6).

For squamous cell carcinoma, TCGA analyzed 178 samples, with mean and median mutation rates of 8.1 and 8.4 mutations/Mb, respectively. Ten genes were significantly altered: TP53 (~80%), CDKN2A, PTEN, PIK3CA, KEAP1, KMT2D (MLL2), HLA-A, NFE2L2, NOTCH1, RB1, involving pathways like CDKN2A/RB1, NFE2L2/KEAP1/CUL3, PI3K/AKT, and SOX2/TP63/NOTCH1 [19]. While mutational testing for squamous NSCLC is less routine, in our 38 squamous cases, we confirmed all TCGA-noted genes except HLA-A (not tested) in >5% at baseline, plus NF1, PRKCI, MTAP, MYC, SOX2, TERC, ARID1A, KRAS, CCND1, ERBB2, FGF12, FGF19, FGF3, KDM6A, and STK11, as well as DNMT3A, ASXL1, TET2, CHEK2, CBL, KMT2D (MLL2), and SF3B1 (all >5%), again indicating possible clonal hematopoiesis. Overall, 94 mutated genes appeared in our squamous cohort (Table 5 and Table S7), revealing multiple potential targets for squamous NSCLC.

### 3.5. Comparison Between Non-Squamous and Squamous NSCLC in Our Series

Overall, squamous tumors exhibited a higher mutation load per patient (median 6.5 vs. 4; average 7.1 vs. 4.8). EGFR was the most frequent driver in non-squamous cases (21% vs. 5% in squamous), followed by KRAS G12C (11% vs. 5%). Together, these two drivers covered 32% of oncogene-driven non-squamous NSCLC. Conversely, NF1 (24%) and PIK3CA (18%) dominated the squamous group, whereas they appeared in only 11% and 5% of the non-squamous group, respectively.

### 3.6. Correlation with Smoking Status

Lung cancers in never-smokers (LCINS) comprise 10–25% of cases, predominantly adenocarcinomas. A recent study of 232 treatment-naive LCINS (Sherlock-Lung) identified three molecular subtypes (piano, mezzo-forte, forte), with an overall mutational burden ~7-fold lower than that in smokers [28]. Genes like EGFR, KRAS, ALK, MET, ERBB2, ROS1, and BRAF were commonly altered, collectively reaching 54.3% [28,29]. In our 26 never-smokers, TP53 was the most frequent (~53.85%), followed by CDKN2A/B, MTAP, SMARCA4, CDK4, KEAP1, MDM2, and RAF1 in >5%. Potential CH genes (DNMT3A, TET2, CBL, JAK2) also appeared in >5%. By contrast, active/former smokers presented more mutations overall (median 6 vs. 3; average 6 vs. 4.5). In that subgroup, NF1 (15%) and PIK3CA (11%) prevailed, whereas never-smokers were driven primarily by EGFR (46%) and, secondarily, NF1 (15%) (Table 5 and Tables S8 and S9).

## 4. Patients and Methods

### 4.1. Study Design

This interventional, prospective, single-center study included stage IV NSCLC patients at the Oncology Institute “Prof. Dr. Ion Chiricuta” in Cluj-Napoca from April 2022 to September 2024, targeting 120 consecutive patients (two-thirds non-squamous histology, one-third squamous). Participants underwent FoundationOne CDx (tissue) and/or FoundationOne Liquid CDx (plasma) plus PD-L1 IHC (SP263) before first-line therapy, with only FoundationOne Liquid CDx being used if tissue was insufficient. At progression, patients were retested with FoundationOne Liquid CDx. Those already tested for EGFR, ALK, and PD-L1 at baseline received only FoundationOne Liquid CDx at progression.

### 4.2. Inclusion and Exclusion Criteria

Patients were eligible if they had a pathologically confirmed diagnosis of stage IV NSCLC, were previously untreated, and either (1) lacked any prior molecular testing and thus qualified for FoundationOne at baseline and progression or (2) had initial single-gene assays (EGFR, ALK, PD-L1) conducted and qualified for FoundationOne testing only at progression. They also had to be ≥18 years of age, sign written informed consent, and be available for follow-up. Exclusion criteria included pregnancy or lactation, a history of other malignancies within 5 years (except for non-melanoma skin cancer or in situ cervical carcinoma), and any prior systemic therapy for advanced/metastatic NSCLC.

### 4.3. NGS Assay and PD-L1 Testing Description

For this study, both FoundationOne^®^ CDx (F1CDx) for tumor tissue and FoundationOne^®^ Liquid CDx (F1LCDx) for plasma were used for comprehensive genomic profiling. F1CDx applies a hybrid-capture, adaptor ligation-based library to DNA from FFPE tumor tissue, assessing 324 genes (base substitutions, indels, copy number alterations, select rearrangements) and reporting microsatellite instability (MSI) and tumor mutational burden (TMB) [6,30]. F1LCDx analyzes circulating cell-free DNA (cfDNA) from blood, evaluating substitutions and indels in 311 genes, copy number alterations in 310 genes, and rearrangements in all 324 genes, as well as blood TMB (bTMB), MSI, and the tumor fraction [6]. Both tests are validated companion diagnostics and serve as broad molecular profiling tools [6]. PD-L1 expression was assessed at baseline using the VENTANA PD-L1 (SP263) IHC assay on FFPE tissue, performed with the OptiView DAB IHC Detection Kit on a VENTANA BenchMark ULTRA instrument [31].

### 4.4. Study Objectives

This study had three main objectives: (1) determine the prevalence of actionable alterations and genomic signatures (TMB, MSI) identified by F1CDx and F1LCDx at baseline and by F1LCDx at progression, recognizing that new driver mutations or immunotherapy biomarkers may emerge over time; (2) compare the number and spectrum of these actionable alterations by tumor histology (non-squamous vs. squamous) and smoking status (active/former vs. never-smokers); and (3) assess the clinical utility of NGS results for personalized treatment, including how often actionable findings lead to targeted therapy and whether barriers (e.g., reimbursement, performance status) impede precision oncology in advanced NSCLC.

### 4.5. Statistical Methods

Analyses were descriptive and exploratory, with no formal hypotheses. The results were tabulated and interpreted descriptively. Categorical variables were presented as counts and percentages and continuous variables using standard descriptive statistics (N, missing, mean, SD, median, min, max). For primary endpoints, absolute and relative frequencies were computed. Where comparisons were made, chi-square tests assessed categorical differences and Student’s *t*-tests compared means, with significance at *p* = 0.05 [32].

## 5. Conclusions

In our study, upfront NGS demonstrated multiple advantages for advanced NSCLC: a fast turnaround (median of 9 days) and a broad panel of druggable mutations, facilitating both standard therapies and clinical trial enrollment. When tissue was unavailable, liquid biopsy still yielded reliable genomic information, and repeating liquid tests at progression provided a dynamic view of evolving targetability. On average, each patient’s distinct mutations decreased from 5.6 at baseline to 4.3 at progression; tissue biopsies had more alterations than liquid biopsies (6 vs. 4.6 per patient), squamous tumors exceeded non-squamous ones (7.1 vs. 4.8), and the number of smokers surpassed that of never-smokers (6 vs. 4.5). Across 119 successfully tested patients, 671 genetic changes were detected in 149 distinct genes, with TP53 being the most frequent (70.59%). Although 74.8% of patients harbored actionable changes (including TMB ≥ 10 mut/Mb), only 35.3% received matched therapy—largely due to poor performance status, reimbursement issues, or a lack of trials. The mutation spectrum varied by histology and smoking. In non-squamous NSCLC (≥5% frequency), EGFR (21%), KRAS G12C (11%), NF1 (11%), and ERBB2 (6%) predominated, whereas squamous tumors featured NF1 (24%), PIK3CA (18%), and ERBB2 (8%). Smokers often showed NF1 (15%), PIK3CA (11%), and KRAS G12C (9%), and never-smokers were driven by EGFR (46%), NF1 (15%), KRAS G12C (8%), BRAF (8%), and MET (8%). No patient was MSI-H, while 26.9% were TMB-high. The PD-L1 IHC results had values < 1% in 33% of patients, 1–49% in 20%, and ≥50% in 18%, and they were unknown in 29%. Overall, integrating TMB with PD-L1 status remains crucial for optimal immunotherapy selection.

## Figures and Tables

**Table 1:** Patient and diagnostic characteristics (*n* = 119).

Category	N	%
Gender		
Male	81	68.1
Female	38	31.9
Age, median (range)	62 (30–86)	
ECOG performance status		
0	10	8.4
1	84	70.6
2	25	21
Smoking status		
Active smoker	33	27.7
Past smoker	56	47.1
Never-smoker	30	25.2
Histology category		
Non-squamous	80	67.2
Squamous	39	32.8
Histology type		
Adenocarcinoma	73	61.3
Large cell	7	5.9
Squamous	38	31.9
Adenosquamous	1	0.8
Type of tissue used and clinical scenario		
Tissue at baseline	43	36.1
Blood at baseline	99	83.2
Blood at progression	34	28.6

**Table 2:** Synopsis of NGS testing results.

Setting	N pts Valid Testing	% pts Tested	Total Muts Found	Distinct Muts	% Mutated Genes/ 324 Tested	Avg Muts/ pt	Range
Tb, Bb, Bp *	119	100	671	149	45.9	5.6	0–16
Tb, Bb *	104	87.4	587	139	42.9	5.6	0–14
Tb	43	36.1	257	98	30.2	6	1–13
Bb	99	83.2	453	113	34.9	4.6	0–11
Bp	34	28.6	146	52	16	4.3	0–10
NSQ (*n* = 80) (Tb, Bb *)	66	82.5	316	100	30.8	4.8	0–12
SQ (*n* = 39) (Tb, Bb *)	38	97.4	271	94	29	7.1	0–14
Smoker (*n* = 89) (Tb, Bb *)	78	87.6	471	127	39.1	6	0–14
Never-smoker (*n* = 30) (Tb, Bb *)	26	21.8	116	57	17.6	4.5	0–10

Abbreviations: Tb—tissue at baseline; Bb—blood at baseline; Bp—blood at progression; muts—mutations; avg—average; SQ—squamous cell carcinoma; NSQ—non-squamous cell carcinoma. * signifies and/or.

**Table 3:** Spectrum and frequency of actionable genetic changes found at baseline and/or progression, as suggested by FoundationOne results.

Genetic Change	N of Pts Harboring Actionable Genetic Changes	% of Pts Harboring Actionable Genetic Changes	Existing Therapeutic Drugs (Examples)
Therapies with clinical relevance in NSCLC			
EGFR	18	15.1	EGFR-TKIs (osimertinib, lazertinib, amivantamab, afatinib, dacomitinib, erlotinib, gefitinib)
KRAS G12C	9	7.6	sotorasib, adagrasib
ALK	3	2.5	lorlatinib, alectinib, brigatinib, ceritinib, crizotinib
MET	2	1.7	capmatinib, tepotinib, telisotuzumab vedotin
RET	1	0.8	selpercatinib, pralsetinib
Subtotal	33	27.7	
Therapies with clinical relevance in both NSCLC and other cancers			
TMB high	32	26.9	Immune check-point inhibitors
ERBB2	7	5.9	trastuzumab deruxtecan
BRAF V600	3	2.5	dabrafenib and trametinib
NTRK	1	0.8	larotrectinib, entrectinib
Subtotal	43	36.1	
Therapies with clinical relevance in other cancers			
NF1	18	15.1	selumetinib, trametinib
PIK3CA	10	8.4	alpelisib
BRCA	4	3.3	PARP-inhibitors (olaparib, niraparib, rucaparib, talazoparib)
PALB2	2	1.7	PARP-inhibitors (olaparib, niraparib, rucaparib, talazoparib)
FGFR2B	1	0.8	bemarituzumab
FBXW7	1	0.8	mTOR-inhibitors (everolimus, temsirolimus)
RAD51	1	0.8	PARP-inhibitors (olaparib, niraparib, rucaparib, talazoparib)
Subtotal	37	31.1	

**Table 4:** Frequency of different actionable gene anomalies and other genes of interest in NGS tissue and liquid testing at baseline and NGS liquid testing at progression.

Gene	F1 Tissue, Baseline		F1 Liquid, Baseline		F1 Liquid, Progression	
	% Gene Alterations (*n* = 257)	% Patients (*n* = 43)	% Gene Alterations (*n* = 453)	% Patients (*n* = 99)	% Gene Alterations (*n* = 146)	% Patients (*n* = 34)
ALK	-	-	0.66	3.03	-	-
BRAF	0.78	4.65	0.66	3.03	1.37	5.88
BRCA1	-	-	0.22	1.01	-	-
BRCA2	0.78	4.65	0.44	2.02	1.37	5.88
EGFR	3.11	18.6	2.87	13.13	2.05	8.82
ERBB2	1.56	9.3	1.1	5.05	0.68	2.94
FGFR2B	-	-	-	-	1.36	5.88
KRAS	3.11	18.6	4.86	22.22	8.22	35.29
MET	0.78	4.65	0.22	1.01	-	-
NF1	2.72	16.28	2.87	13.13	3.42	14.71
NTRK	-	-	-	-	0.68	2.94
PALB2	-	-	0.44	2.02	-	-
PIK3CA	2.72	16.28	1.32	6.06	0.68	2.94
RAD51	-	-	0.22	1.01	-	-
RET	0.39	2.33	-	-	-	-
TP53	12.06	72.09	15.01	68.69	16.44	70.59
KEAP1	1.95	11.63	1.99	9.09	4.11	17.65
STK11	3.11	18.6	3.09	14.14	4.79	20.59
SMARCA4	0.78	4.65	0.44	2.02	-	-

**Table 5:** Comparison of main genetic changes found in >10% patients for different subsets of patients in descending order of frequencies.

	Baseline	Progression
# of Patients	104	34
Most frequently mutated genes (>10% pts)	TP53, KRAS, CDKN2A/B, STK11, EGFR, NF1, KEAP1	TP53, KRAS, STK11, KEAP1, NF1, CDKN2A
Changes in genes that could represent CH	DNMT3A, TET2, ASXL1	DNMT3A, ASXL1, ATM, CHEK2, TET2
	Squamous	Non-Squamous
# of Patients at baseline	38	66
Most frequently mutated genes (TCGA Landmark Papers)	TP53, CDKN2A, PTEN, PIK3CA, KEAP1	TP53, KRAS, EGFR, BRAF
Most frequently mutated genes (>10% pts)	TP53, CDKN2A/B, NF1, PIK3CA, PTEN, PRKCI, KEAP1, MTAP, MYC, NOTCH1, SOX2, TERC, ARID1A, KRAS	TP53, KRAS, STK11, EGFR, CDKN2A/B, KEAP1, NF1.
Changes in genes that could represent CH	DNMT3A, ASXL1, TET2, CHEK2	DNMT3A, TET2, ASXL1, ATM
	Smokers/Former smokers	Never-Smokers
# of Patients at baseline	78	26
Most frequently mutated genes (>10% pts)	TP53, KRAS, STK11, CDKN2A/B, NF1, KEAP1, PIK3CA, RB1	TP53, EGFR, CDKN2A/B, NF1, KRAS, MTAP, SMARCA4
Changes in genes that could represent CH	DNMT3A, ASXL1, TET2, ATM, CHEK2	DNMT3A, TET2 and CBL

## Data Availability

The raw data supporting the conclusions of this article will be made available by the authors on request.

## Supplementary Materials

The following supporting information can be downloaded at: https://www.mdpi.com/article/10.3390/ijms26073403/s1.
