# Supplementary material for: Prospective Upfront Next-Generation Sequencing for Advanced Non-Small Cell Lung Cancer: Real-World Outcomes from the Ion Chiricuță Oncology Institute
Source: Int J Mol Sci. 2025 Apr 5;26(7):3403. doi: 10.3390/ijms26073403 (PMC11989902; doi:10.3390/ijms26073403)
Supplement: Supplementary file 1 [file ijms-26-03403-s001.zip › ijms-3559836-supplementary.pdf]

Table S1. Genetic abnormalities found overall (F1CDx Tissue at baseline, F1LCDx Blood at baseline, F1LCDx Blood at progression)

	Gene, alphabetical	N muts	% genes (n=671)	% pts (n=119)	Gene, descending frequency	N muts	% genes (n=671)	% pts (n=119)
1	ACVR1B	1	0,15%	0,84%	TP53	84	12,52%	70,59%
2	AKT1	1	0,15%	0,84%	DNMT3A	40	5,96%	33,61%
3	AKT2	1	0,15%	0,84%	KRAS	32	4,77%	26,89%
4	ALK	3	0,45%	2,52%	STK11	23	3,43%	19,33%
5	APC	7	1,04%	5,88%	TET2	23	3,43%	19,33%
6	ARAF	2	0,30%	1,68%	CDKN2A/B	21	3,13%	17,65%
7	ARFRP1	1	0,15%	0,84%	ASXL1	19	2,83%	15,97%
8	ARID1A	6	0,89%	5,04%	EGFR	18	2,68%	15,13%
9	ASXL1	19	2,83%	15,97%	NF1	18	2,68%	15,13%
10	ATM	14	2,09%	11,76%	ATM	14	2,09%	11,76%
11	AURKA	1	0,15%	0,84%	CHEK2	14	2,09%	11,76%
12	AURKB	1	0,15%	0,84%	KEAP1	14	2,09%	11,76%
13	BARD1	1	0,15%	0,84%	MTAP	10	1,49%	8,40%
14	BARD1	1	0,15%	0,84%	PIK3CA	10	1,49%	8,40%
15	BCL2L1	1	0,15%	0,84%	JAK2	9	1,34%	7,56%
16	BCOR	6	0,89%	5,04%	RB1	9	1,34%	7,56%
17	BCORL1	2	0,30%	1,68%	CBL	8	1,19%	6,72%
18	BRAF	7	1,04%	5,88%	MYC	8	1,19%	6,72%
19	BRCA1	1	0,15%	0,84%	PTEN	8	1,19%	6,72%
20	BRCA2	2	0,30%	1,68%	SF3B1	8	1,19%	6,72%
21	CARD11	1	0,15%	0,84%	APC	7	1,04%	5,88%
22	CBL	8	1,19%	6,72%	BRAF	7	1,04%	5,88%
23	CCND1	3	0,45%	2,52%	ERBB2	7	1,04%	5,88%
24	CCNE	1	0,15%	0,84%	NOTCH1	7	1,04%	5,88%
25	CCNE1	2	0,30%	1,68%	RBM10	7	1,04%	5,88%
26	CDH1	1	0,15%	0,84%	ARID1A	6	0,89%	5,04%
27	CDK12	2	0,30%	1,68%	BCOR	6	0,89%	5,04%
28	CDK4	3	0,45%	2,52%	KDM6A	6	0,89%	5,04%
29	CDK6	1	0,15%	0,84%	KMT2D	6	0,89%	5,04%
30	CDKN1B	1	0,15%	0,84%	PRKCI	6	0,89%	5,04%
31	CDKN21/b	1	0,15%	0,84%	SOX2	5	0,75%	4,20%
32	CDKN2A/B	21	3,13%	17,65%	TERC	5	0,75%	4,20%
33	CDKN2C	1	0,15%	0,84%	CREBBP	4	0,60%	3,36%
34	CHEK2	14	2,09%	11,76%	GNAS	4	0,60%	3,36%
35	CIC	1	0,15%	0,84%	NKX2-1	4	0,60%	3,36%
36	CREBBP	4	0,60%	3,36%	NSD3 (WHSC1L1)	4	0,60%	3,36%
37	CRKL	2	0,30%	1,68%	PTPN11	4	0,60%	3,36%
38	CTCF	2	0,30%	1,68%	RAD21	4	0,60%	3,36%
39	CTNNA1	1	0,15%	0,84%	SETD2	4	0,60%	3,36%

40	CTNNB1	2	0,30%	1,68%	ALK	3	0,45%	2,52%
41	CUL3	3	0,45%	2,52%	CCND1	3	0,45%	2,52%
42	DKN2A/B	1	0,15%	0,84%	CDK4	3	0,45%	2,52%
43	DNMT3A	40	5,96%	33,61%	CUL3	3	0,45%	2,52%
44	EGFR	18	2,68%	15,13%	EP300	3	0,45%	2,52%
45	EP300	3	0,45%	2,52%	FANCL	3	0,45%	2,52%
46	EPHA3	1	0,15%	0,84%	FGF12	3	0,45%	2,52%
47	ERBB2	7	1,04%	5,88%	FGF19	3	0,45%	2,52%
48	EZH2	1	0,15%	0,84%	FGF3	3	0,45%	2,52%
49	FANCA	2	0,30%	1,68%	FGFR1	3	0,45%	2,52%
50	FANCC	1	0,15%	0,84%	MDM2	3	0,45%	2,52%
51	FANCG	1	0,15%	0,84%	MEN1	3	0,45%	2,52%
52	FANCL	3	0,45%	2,52%	MYCL1	3	0,45%	2,52%
53	FBXW7	2	0,30%	1,68%	NFE2L2	3	0,45%	2,52%
54	FGF10	2	0,30%	1,68%	NFKBIA	3	0,45%	2,52%
55	FGF12	3	0,45%	2,52%	SMARCA4	3	0,45%	2,52%
56	FGF19	3	0,45%	2,52%	SPEN	3	0,45%	2,52%
57	FGF3	3	0,45%	2,52%	STAG2	3	0,45%	2,52%
58	FGF4	2	0,30%	1,68%	TERT	3	0,45%	2,52%
59	FGFR1	3	0,45%	2,52%	ARAF	2	0,30%	1,68%
60	FGFR2	1	0,15%	0,84%	BCORL1	2	0,30%	1,68%
61	FGFR2B	1	0,15%	0,84%	BRCA2	2	0,30%	1,68%
62	FGFR3	2	0,30%	1,68%	CCNE1	2	0,30%	1,68%
63	FUBP1	1	0,15%	0,84%	CDK12	2	0,30%	1,68%
64	GATA4	1	0,15%	0,84%	CRKL	2	0,30%	1,68%
65	GNAS	4	0,60%	3,36%	CTCF	2	0,30%	1,68%
66	HGF	1	0,15%	0,84%	CTNNB1	2	0,30%	1,68%
67	HSD3B1	2	0,30%	1,68%	FANCA	2	0,30%	1,68%
68	IDH1	1	0,15%	0,84%	FBXW7	2	0,30%	1,68%
69	IDH2	2	0,30%	1,68%	FGF10	2	0,30%	1,68%
70	IRF2	2	0,30%	1,68%	FGF4	2	0,30%	1,68%
71	JAK2	9	1,34%	7,56%	FGFR3	2	0,30%	1,68%
72	JUN	1	0,15%	0,84%	HSD3B1	2	0,30%	1,68%
73	KDM5A	2	0,30%	1,68%	IDH2	2	0,30%	1,68%
74	KDM5C	1	0,15%	0,84%	IRF2	2	0,30%	1,68%
75	KDM6A	6	0,89%	5,04%	KDM5A	2	0,30%	1,68%
76	KEAP1	14	2,09%	11,76%	MAP2K4	2	0,30%	1,68%
77	KEL	1	0,15%	0,84%	MET	2	0,30%	1,68%
78	KMT2D	6	0,89%	5,04%	NBN	2	0,30%	1,68%
79	KRAS	32	4,77%	26,89%	NF2	2	0,30%	1,68%
80	MAP2K1 (MEK)	1	0,15%	0,84%	NOTCH2	2	0,30%	1,68%
81	MAP2K4	2	0,30%	1,68%	PALB2	2	0,30%	1,68%
82	MAPK1	1	0,15%	0,84%	PBRM1	2	0,30%	1,68%
83	MDM2	3	0,45%	2,52%	PDGFRA	2	0,30%	1,68%

84	MEN1	3	0,45%	2,52%	PIK3R1	2	0,30%	1,68%
85	MET	2	0,30%	1,68%	RAF1	2	0,30%	1,68%
86	MLL2	1	0,15%	0,84%	REL	2	0,30%	1,68%
87	MSH6	1	0,15%	0,84%	SMAD4	2	0,30%	1,68%
88	MTAP	10	1,49%	8,40%	TSC2	2	0,30%	1,68%
89	MTOR	1	0,15%	0,84%	ACVR1B	1	0,15%	0,84%
90	MYC	8	1,19%	6,72%	AKT1	1	0,15%	0,84%
91	MYCL1	3	0,45%	2,52%	AKT2	1	0,15%	0,84%
92	NBN	2	0,30%	1,68%	ARFRP1	1	0,15%	0,84%
93	NF1	18	2,68%	15,13%	AURKA	1	0,15%	0,84%
94	NF2	2	0,30%	1,68%	AURKB	1	0,15%	0,84%
95	NFE2L2	3	0,45%	2,52%	BARD1	1	0,15%	0,84%
96	NFKBIA	3	0,45%	2,52%	BARD1	1	0,15%	0,84%
97	NKX2-1	4	0,60%	3,36%	BCL2L1	1	0,15%	0,84%
98	NOTCH1	7	1,04%	5,88%	BRCA1	1	0,15%	0,84%
99	NOTCH2	2	0,30%	1,68%	CARD11	1	0,15%	0,84%
100	NRAS	1	0,15%	0,84%	CCNE	1	0,15%	0,84%
101	NSD3 (WHSC1L1)	4	0,60%	3,36%	CDH1	1	0,15%	0,84%
102	NTRK1	1	0,15%	0,84%	CDK6	1	0,15%	0,84%
103	PALB2	2	0,30%	1,68%	CDKN1B	1	0,15%	0,84%
104	PAX5	1	0,15%	0,84%	CDKN21/b	1	0,15%	0,84%
105	PBRM1	2	0,30%	1,68%	CDKN2C	1	0,15%	0,84%
106	PDCD1	1	0,15%	0,84%	CIC	1	0,15%	0,84%
107	PDCD1LG2	1	0,15%	0,84%	CTNNA1	1	0,15%	0,84%
108	PDGFRA	2	0,30%	1,68%	DKN2A/B	1	0,15%	0,84%
109	PDGFRB	1	0,15%	0,84%	EPHA3	1	0,15%	0,84%
110	PIK3CA	10	1,49%	8,40%	EZH2	1	0,15%	0,84%
111	PIK3R1	2	0,30%	1,68%	FANCC	1	0,15%	0,84%
112	PMS2	1	0,15%	0,84%	FANCG	1	0,15%	0,84%
113	PPP2R2A	1	0,15%	0,84%	FGFR2	1	0,15%	0,84%
114	PRDM1	1	0,15%	0,84%	FGFR2B	1	0,15%	0,84%
115	PRKCI	6	0,89%	5,04%	FUBP1	1	0,15%	0,84%
116	PTEN	8	1,19%	6,72%	GATA4	1	0,15%	0,84%
117	PTPN11	4	0,60%	3,36%	HGF	1	0,15%	0,84%
118	PTPRO	1	0,15%	0,84%	IDH1	1	0,15%	0,84%
119	QKI	1	0,15%	0,84%	JUN	1	0,15%	0,84%
120	RAD21	4	0,60%	3,36%	KDM5C	1	0,15%	0,84%
121	RAD51C	1	0,15%	0,84%	KEL	1	0,15%	0,84%
122	RAD54L	1	0,15%	0,84%	MAP2K1 (MEK)	1	0,15%	0,84%
123	RAF1	2	0,30%	1,68%	MAPK1	1	0,15%	0,84%
124	RB1	9	1,34%	7,56%	MLL2	1	0,15%	0,84%
125	RBM10	7	1,04%	5,88%	MSH6	1	0,15%	0,84%
126	REL	2	0,30%	1,68%	MTOR	1	0,15%	0,84%
127	RET	1	0,15%	0,84%	NRAS	1	0,15%	0,84%

128	RICTOR	1	0,15%	0,84%	NTRK1	1	0,15%	0,84%
129	RNF43	1	0,15%	0,84%	PAX5	1	0,15%	0,84%
130	SETD2	4	0,60%	3,36%	PDCD1	1	0,15%	0,84%
131	SF3B1	8	1,19%	6,72%	PDCD1LG2	1	0,15%	0,84%
132	SMAD4	2	0,30%	1,68%	PDGFRB	1	0,15%	0,84%
133	SMARCA4	3	0,45%	2,52%	PMS2	1	0,15%	0,84%
134	SMARCB1	1	0,15%	0,84%	PPP2R2A	1	0,15%	0,84%
135	SOX2	5	0,75%	4,20%	PRDM1	1	0,15%	0,84%
136	SPEN	3	0,45%	2,52%	PTPRO	1	0,15%	0,84%
137	SPOP	1	0,15%	0,84%	QKI	1	0,15%	0,84%
138	STAG2	3	0,45%	2,52%	RAD51C	1	0,15%	0,84%
139	STAT3	1	0,15%	0,84%	RAD54L	1	0,15%	0,84%
140	STK11	23	3,43%	19,33%	RET	1	0,15%	0,84%
141	TEK	1	0,15%	0,84%	RICTOR	1	0,15%	0,84%
142	TERC	5	0,75%	4,20%	RNF43	1	0,15%	0,84%
143	TERT	3	0,45%	2,52%	SMARCB1	1	0,15%	0,84%
144	TET2	23	3,43%	19,33%	SPOP	1	0,15%	0,84%
145	TP53	84	12,52%	70,59%	STAT3	1	0,15%	0,84%
146	TSC2	2	0,30%	1,68%	TEK	1	0,15%	0,84%
147	U2AF1	1	0,15%	0,84%	U2AF1	1	0,15%	0,84%
148	XRCC2	1	0,15%	0,84%	XRCC2	1	0,15%	0,84%
149	ZNF703	1	0,15%	0,84%	ZNF703	1	0,15%	0,84%
150	No mut (4 pts)	0	0,00%	0,00%	No mut (4 pts)	0	0,00%	0,00%
	Total	671	100,00%		Total	671	100,00%	

Total number of patients tested n=119. Total number of genetic abnormalities found n=671. Genes in red may represent clonal hematopoesis.

Table S2. Genetic abnormalities found at baseline (F1CDx Tissue at baseline, F1LCDx Blood at baseline)

	Gene, alphabetical	N muts	% genes (n=587)	% pts (n=104)
1	AKT1	1	0,17%	0,96%
2	AKT2	1	0,17%	0,96%
3	ALK	3	0,51%	2,88%
4	APC	5	0,85%	4,81%
5	ARAF	2	0,34%	1,92%
6	ARFRP1	1	0,17%	0,96%
7	ARID1A	6	1,02%	5,77%
8	ASXL1	16	2,73%	15,38%
9	ATM	9	1,53%	8,65%
10	AURKA	1	0,17%	0,96%
11	AURKB	1	0,17%	0,96%
12	BARD1	1	0,17%	0,96%
13	BCL2L1	1	0,17%	0,96%
14	BCOR	6	1,02%	5,77%
15	BCORL1	2	0,34%	1,92%
16	BRAF	5	0,85%	4,81%
17	BRCA1	1	0,17%	0,96%
18	BRCA2	2	0,34%	1,92%
19	CARD11	1	0,17%	0,96%
20	CBL	6	1,02%	5,77%
21	CCND1	3	0,51%	2,88%
22	CCNE	1	0,17%	0,96%
23	CCNE1	2	0,34%	1,92%
24	CDH1	1	0,17%	0,96%
25	CDK12	2	0,34%	1,92%
26	CDK4	3	0,51%	2,88%
27	CDK6	1	0,17%	0,96%
28	CDKN1B	1	0,17%	0,96%
29	CDKN21/b	1	0,17%	0,96%
30	CDKN2A/B	20	3,41%	19,23%
31	CDKN2C	1	0,17%	0,96%
32	CHEK2	9	1,53%	8,65%
33	CIC	1	0,17%	0,96%
34	CREBBP	3	0,51%	2,88%
35	CRKL	2	0,34%	1,92%
36	CTCF	2	0,34%	1,92%
37	CTNNA1	1	0,17%	0,96%
38	CTNNB1	2	0,34%	1,92%
39	CUL3	3	0,51%	2,88%
40	DKN2A/B	1	0,17%	0,96%

	Gene, descending frequency	N muts	% genes (n=587)	% pts (n=104)
1	TP53	72	12,27%	69,23%
2	DNMT3A	33	5,62%	31,73%
3	KRAS	26	4,43%	25,00%
4	CDKN2A/B	20	3,41%	19,23%
5	TET2	19	3,24%	18,27%
6	STK11	18	3,07%	17,31%
7	ASXL1	16	2,73%	15,38%
8	EGFR	16	2,73%	15,38%
9	NF1	16	2,73%	15,38%
10	KEAP1	12	2,04%	11,54%
11	MTAP	10	1,70%	9,62%
12	PIK3CA	10	1,70%	9,62%
13	ATM	9	1,53%	8,65%
14	CHEK2	9	1,53%	8,65%
15	RB1	9	1,53%	8,65%
16	PTEN	8	1,36%	7,69%
17	ERBB2	7	1,19%	6,73%
18	JAK2	7	1,19%	6,73%
19	MYC	7	1,19%	6,73%
20	NOTCH1	7	1,19%	6,73%
21	SF3B1	7	1,19%	6,73%
22	ARID1A	6	1,02%	5,77%
23	BCOR	6	1,02%	5,77%
24	CBL	6	1,02%	5,77%
25	KDM6A	6	1,02%	5,77%
26	KMT2D	6	1,02%	5,77%
27	PRKCI	6	1,02%	5,77%
28	RBM10	6	1,02%	5,77%
29	APC	5	0,85%	4,81%
30	BRAF	5	0,85%	4,81%
31	SOX2	5	0,85%	4,81%
32	TERC	5	0,85%	4,81%
33	NKX2-1	4	0,68%	3,85%
34	NSD3 (WHSC1L1)	4	0,68%	3,85%
35	PTPN11	4	0,68%	3,85%
36	ALK	3	0,51%	2,88%
37	CCND1	3	0,51%	2,88%
38	CDK4	3	0,51%	2,88%
39	CREBBP	3	0,51%	2,88%
40	CUL3	3	0,51%	2,88%

41	DNMT3A	33	5,62%	31,73%	41	EP300	3	0,51%	2,88%
42	EGFR	16	2,73%	15,38%	42	FANCL	3	0,51%	2,88%
43	EP300	3	0,51%	2,88%	43	FGF12	3	0,51%	2,88%
44	EPHA3	1	0,17%	0,96%	44	FGF19	3	0,51%	2,88%
45	ERBB2	7	1,19%	6,73%	45	FGF3	3	0,51%	2,88%
46	EZH2	1	0,17%	0,96%	46	FGFR1	3	0,51%	2,88%
47	FANCA	2	0,34%	1,92%	47	GNAS	3	0,51%	2,88%
48	FANCC	1	0,17%	0,96%	48	MDM2	3	0,51%	2,88%
49	FANCL	3	0,51%	2,88%	49	MYCL1	3	0,51%	2,88%
50	FBXW7	2	0,34%	1,92%	50	NFE2L2	3	0,51%	2,88%
51	FGF10	2	0,34%	1,92%	51	SETD2	3	0,51%	2,88%
52	FGF12	3	0,51%	2,88%	52	SMARCA4	3	0,51%	2,88%
53	FGF19	3	0,51%	2,88%	53	SPEN	3	0,51%	2,88%
54	FGF3	3	0,51%	2,88%	54	STAG2	3	0,51%	2,88%
55	FGF4	2	0,34%	1,92%	55	TERT	3	0,51%	2,88%
56	FGFR1	3	0,51%	2,88%	56	ARAF	2	0,34%	1,92%
57	FGFR3	2	0,34%	1,92%	57	BCORL1	2	0,34%	1,92%
58	FUBP1	1	0,17%	0,96%	58	BRCA2	2	0,34%	1,92%
59	GATA4	1	0,17%	0,96%	59	CCNE1	2	0,34%	1,92%
60	GNAS	3	0,51%	2,88%	60	CDK12	2	0,34%	1,92%
61	HGF	1	0,17%	0,96%	61	CRKL	2	0,34%	1,92%
62	HSD3B1	2	0,34%	1,92%	62	CTCF	2	0,34%	1,92%
63	IDH1	1	0,17%	0,96%	63	CTNNB1	2	0,34%	1,92%
64	IDH2	2	0,34%	1,92%	64	FANCA	2	0,34%	1,92%
65	IRF2	1	0,17%	0,96%	65	FBXW7	2	0,34%	1,92%
66	JAK2	7	1,19%	6,73%	66	FGF10	2	0,34%	1,92%
67	JUN	1	0,17%	0,96%	67	FGF4	2	0,34%	1,92%
68	KDM5A	2	0,34%	1,92%	68	FGFR3	2	0,34%	1,92%
69	KDM5C	1	0,17%	0,96%	69	HSD3B1	2	0,34%	1,92%
70	KDM6A	6	1,02%	5,77%	70	IDH2	2	0,34%	1,92%
71	KEAP1	12	2,04%	11,54%	71	KDM5A	2	0,34%	1,92%
72	KEL	1	0,17%	0,96%	72	MAP2K4	2	0,34%	1,92%
73	KMT2D	6	1,02%	5,77%	73	MET	2	0,34%	1,92%
74	KRAS	26	4,43%	25,00%	74	NBN	2	0,34%	1,92%
75	MAP2K4	2	0,34%	1,92%	75	NF2	2	0,34%	1,92%
76	MAPK1	1	0,17%	0,96%	76	NFKBIA	2	0,34%	1,92%
77	MDM2	3	0,51%	2,88%	77	NOTCH2	2	0,34%	1,92%
78	MEN1	1	0,17%	0,96%	78	PALB2	2	0,34%	1,92%
79	MET	2	0,34%	1,92%	79	PBRM1	2	0,34%	1,92%
80	MLL2	1	0,17%	0,96%	80	PDGFRA	2	0,34%	1,92%
81	MSH6	1	0,17%	0,96%	81	PIK3R1	2	0,34%	1,92%
82	MTAP	10	1,70%	9,62%	82	RAD21	2	0,34%	1,92%
83	MTOR	1	0,17%	0,96%	83	RAF1	2	0,34%	1,92%
84	MYC	7	1,19%	6,73%	84	REL	2	0,34%	1,92%

85	MYCL1	3	0,51%	2,88%	85	SMAD4	2	0,34%	1,92%
86	NBN	2	0,34%	1,92%	86	TSC2	2	0,34%	1,92%
87	NF1	16	2,73%	15,38%	87	AKT1	1	0,17%	0,96%
88	NF2	2	0,34%	1,92%	88	AKT2	1	0,17%	0,96%
89	NFE2L2	3	0,51%	2,88%	89	ARFRP1	1	0,17%	0,96%
90	NFKBIA	2	0,34%	1,92%	90	AURKA	1	0,17%	0,96%
91	NKX2-1	4	0,68%	3,85%	91	AURKB	1	0,17%	0,96%
92	NOTCH1	7	1,19%	6,73%	92	BARD1	1	0,17%	0,96%
93	NOTCH2	2	0,34%	1,92%	93	BCL2L1	1	0,17%	0,96%
94	NRAS	1	0,17%	0,96%	94	BRCA1	1	0,17%	0,96%
95	NSD3 (WHSC1L1)	4	0,68%	3,85%	95	CARD11	1	0,17%	0,96%
96	PALB2	2	0,34%	1,92%	96	CCNE	1	0,17%	0,96%
97	PAX5	1	0,17%	0,96%	97	CDH1	1	0,17%	0,96%
98	PBRM1	2	0,34%	1,92%	98	CDK6	1	0,17%	0,96%
99	PDCD1	1	0,17%	0,96%	99	CDKN1B	1	0,17%	0,96%
100	PDCD1LG2	1	0,17%	0,96%	100	CDKN21/b	1	0,17%	0,96%
101	PDGFRA	2	0,34%	1,92%	101	CDKN2C	1	0,17%	0,96%
102	PIK3CA	10	1,70%	9,62%	102	CIC	1	0,17%	0,96%
103	PIK3R1	2	0,34%	1,92%	103	CTNNA1	1	0,17%	0,96%
104	PMS2	1	0,17%	0,96%	104	DKN2A/B	1	0,17%	0,96%
105	PRDM1	1	0,17%	0,96%	105	EPHA3	1	0,17%	0,96%
106	PRKCI	6	1,02%	5,77%	106	EZH2	1	0,17%	0,96%
107	PTEN	8	1,36%	7,69%	107	FANCC	1	0,17%	0,96%
108	PTPN11	4	0,68%	3,85%	108	FUBP1	1	0,17%	0,96%
109	QKI	1	0,17%	0,96%	109	GATA4	1	0,17%	0,96%
110	RAD21	2	0,34%	1,92%	110	HGF	1	0,17%	0,96%
111	RAD51C	1	0,17%	0,96%	111	IDH1	1	0,17%	0,96%
112	RAD54L	1	0,17%	0,96%	112	IRF2	1	0,17%	0,96%
113	RAF1	2	0,34%	1,92%	113	JUN	1	0,17%	0,96%
114	RB1	9	1,53%	8,65%	114	KDM5C	1	0,17%	0,96%
115	RBM10	6	1,02%	5,77%	115	KEL	1	0,17%	0,96%
116	REL	2	0,34%	1,92%	116	MAPK1	1	0,17%	0,96%
117	RET	1	0,17%	0,96%	117	MEN1	1	0,17%	0,96%
118	RICTOR	1	0,17%	0,96%	118	MLL2	1	0,17%	0,96%
119	RNF43	1	0,17%	0,96%	119	MSH6	1	0,17%	0,96%
120	SETD2	3	0,51%	2,88%	120	MTOR	1	0,17%	0,96%
121	SF3B1	7	1,19%	6,73%	121	NRAS	1	0,17%	0,96%
122	SMAD4	2	0,34%	1,92%	122	PAX5	1	0,17%	0,96%
123	SMARCA4	3	0,51%	2,88%	123	PDCD1	1	0,17%	0,96%
124	SMARCB1	1	0,17%	0,96%	124	PDCD1LG2	1	0,17%	0,96%
125	SOX2	5	0,85%	4,81%	125	PMS2	1	0,17%	0,96%
126	SPEN	3	0,51%	2,88%	126	PRDM1	1	0,17%	0,96%
127	SPOP	1	0,17%	0,96%	127	QKI	1	0,17%	0,96%
128	STAG2	3	0,51%	2,88%	128	RAD51C	1	0,17%	0,96%

129	STAT3	1	0,17%	0,96%
130	STK11	18	3,07%	17,31%
131	TEK	1	0,17%	0,96%
132	TERC	5	0,85%	4,81%
133	TERT	3	0,51%	2,88%
134	TET2	19	3,24%	18,27%
135	TP53	72	12,27%	69,23%
136	TSC2	2	0,34%	1,92%
137	U2AF1	1	0,17%	0,96%
138	XRCC2	1	0,17%	0,96%
139	ZNF703	1	0,17%	0,96%
140	No mut (3 pts)	0	0,00%	0,00%
	Total	587	100,00 %	

129	RAD54L	1	0,17%	0,96%
130	RET	1	0,17%	0,96%
131	RICTOR	1	0,17%	0,96%
132	RNF43	1	0,17%	0,96%
133	SMARCB1	1	0,17%	0,96%
134	SPOP	1	0,17%	0,96%
135	STAT3	1	0,17%	0,96%
136	TEK	1	0,17%	0,96%
137	U2AF1	1	0,17%	0,96%
138	XRCC2	1	0,17%	0,96%
139	ZNF703	1	0,17%	0,96%
140	No mut (3 pts)	0	0,00%	0,00%
	Total	587	100%	

Total number of patients tested N=104. Total number of genetic abnormalities found n=587. Genes in red may represent clonal hematopoesis.

Table S3. Genetic abnormalities found at baseline in tissue biopsy (F1CDx Tissue)

	Gene, alphabetical	N muts	% genes (n=257)	% pts (n=43)		Gene, descending frequency	N nuts	% genes (n=257)	% pts (n=43)
1	AKT1	1	0,39%	2,33%	1	TP53	31	12,06%	72,09%
2	APC	1	0,39%	2,33%	2	CDKN2A/B	16	6,23%	37,21%
3	ARAF	1	0,39%	2,33%	3	MTAP	10	3,89%	23,26%
4	ARID1A	3	1,17%	6,98%	4	EGFR	8	3,11%	18,60%
5	ATM	1	0,39%	2,33%	5	KRAS	8	3,11%	18,60%
6	AURKB	1	0,39%	2,33%	6	STK11	8	3,11%	18,60%
7	BCL2L1	1	0,39%	2,33%	7	NF1	7	2,72%	16,28%
8	BCOR	2	0,78%	4,65%	8	PIK3CA	7	2,72%	16,28%
9	BCORL1	1	0,39%	2,33%	9	PRKCI	6	2,33%	13,95%
10	BRAF	2	0,78%	4,65%	10	PTEN	6	2,33%	13,95%
11	BRCA2	2	0,78%	4,65%	11	KEAP1	5	1,95%	11,63%
12	CARD11	1	0,39%	2,33%	12	SOX2	5	1,95%	11,63%
13	CBL	1	0,39%	2,33%	13	TERC	5	1,95%	11,63%
14	CCND1	2	0,78%	4,65%	14	ERBB2	4	1,56%	9,30%
15	CCNE	1	0,39%	2,33%	15	KMT2D	4	1,56%	9,30%
16	CCNE1	1	0,39%	2,33%	16	MYC	4	1,56%	9,30%
17	CDK12	1	0,39%	2,33%	17	RB1	4	1,56%	9,30%
18	CDK4	2	0,78%	4,65%	18	ARID1A	3	1,17%	6,98%
19	CDK6	1	0,39%	2,33%	19	FGF12	3	1,17%	6,98%
20	CDKN1B	1	0,39%	2,33%	20	MYCL1	3	1,17%	6,98%
21	CDKN2A/B	16	6,23%	37,21%	21	NKX2-1	3	1,17%	6,98%
22	CHEK2	1	0,39%	2,33%	22	NOTCH1	3	1,17%	6,98%
23	CIC	1	0,39%	2,33%	23	RBM10	3	1,17%	6,98%
24	CRKL	2	0,78%	4,65%	24	SPEN	3	1,17%	6,98%
25	CTNNB1	1	0,39%	2,33%	25	BCOR	2	0,78%	4,65%
26	CUL3	1	0,39%	2,33%	26	BRAF	2	0,78%	4,65%
27	DKN2A/B	1	0,39%	2,33%	27	BRCA2	2	0,78%	4,65%
28	EGFR	8	3,11%	18,60%	28	CCND1	2	0,78%	4,65%
29	EP300	2	0,78%	4,65%	29	CDK4	2	0,78%	4,65%
30	ERBB2	4	1,56%	9,30%	30	CRKL	2	0,78%	4,65%
31	FANCA	1	0,39%	2,33%	31	EP300	2	0,78%	4,65%
32	FANCC	1	0,39%	2,33%	32	FANCL	2	0,78%	4,65%
33	FANCL	2	0,78%	4,65%	33	FBXW7	2	0,78%	4,65%
34	FBXW7	2	0,78%	4,65%	34	FGF10	2	0,78%	4,65%
35	FGF10	2	0,78%	4,65%	35	FGF19	2	0,78%	4,65%
36	FGF12	3	1,17%	6,98%	36	FGF3	2	0,78%	4,65%
37	FGF19	2	0,78%	4,65%	37	KDM5A	2	0,78%	4,65%
38	FGF3	2	0,78%	4,65%	38	KDM6A	2	0,78%	4,65%
39	FGF4	1	0,39%	2,33%	39	MAP2K4	2	0,78%	4,65%
40	FGFR1	1	0,39%	2,33%	40	MDM2	2	0,78%	4,65%

41	FGFR3	1	0,39%	2,33%	41	MET	2	0,78%	4,65%
42	FUBP1	1	0,39%	2,33%	42	NF2	2	0,78%	4,65%
43	GATA4	1	0,39%	2,33%	43	NFE2L2	2	0,78%	4,65%
44	HGF	1	0,39%	2,33%	44	NFKBIA	2	0,78%	4,65%
45	HSD3B1	1	0,39%	2,33%	45	NOTCH2	2	0,78%	4,65%
46	JAK2	1	0,39%	2,33%	46	NSD3 (WHSC1L1)	2	0,78%	4,65%
47	JUN	1	0,39%	2,33%	47	SMARCA4	2	0,78%	4,65%
48	KDM5A	2	0,78%	4,65%	48	TET2	2	0,78%	4,65%
49	KDM6A	2	0,78%	4,65%	49	AKT1	1	0,39%	2,33%
50	KEAP1	5	1,95%	11,63%	50	APC	1	0,39%	2,33%
51	KEL	1	0,39%	2,33%	51	ARAF	1	0,39%	2,33%
52	KMT2D	4	1,56%	9,30%	52	ATM	1	0,39%	2,33%
53	KRAS	8	3,11%	18,60%	53	AURKB	1	0,39%	2,33%
54	MAP2K4	2	0,78%	4,65%	54	BCL2L1	1	0,39%	2,33%
55	MAPK1	1	0,39%	2,33%	55	BCORL1	1	0,39%	2,33%
56	MDM2	2	0,78%	4,65%	56	CARD11	1	0,39%	2,33%
57	MET	2	0,78%	4,65%	57	CBL	1	0,39%	2,33%
58	MTAP	10	3,89%	23,26%	58	CCNE	1	0,39%	2,33%
59	MTOR	1	0,39%	2,33%	59	CCNE1	1	0,39%	2,33%
60	MYC	4	1,56%	9,30%	60	CDK12	1	0,39%	2,33%
61	MYCL1	3	1,17%	6,98%	61	CDK6	1	0,39%	2,33%
62	NF1	7	2,72%	16,28%	62	CDKN1B	1	0,39%	2,33%
63	NF2	2	0,78%	4,65%	63	CHEK2	1	0,39%	2,33%
64	NFE2L2	2	0,78%	4,65%	64	CIC	1	0,39%	2,33%
65	NFKBIA	2	0,78%	4,65%	65	CTNNB1	1	0,39%	2,33%
66	NKX2-1	3	1,17%	6,98%	66	CUL3	1	0,39%	2,33%
67	NOTCH1	3	1,17%	6,98%	67	DKN2A/B	1	0,39%	2,33%
68	NOTCH2	2	0,78%	4,65%	68	FANCA	1	0,39%	2,33%
69	NSD3 (WHSC1L1)	2	0,78%	4,65%	69	FANCC	1	0,39%	2,33%
70	PAX5	1	0,39%	2,33%	70	FGF4	1	0,39%	2,33%
71	PDCD1	1	0,39%	2,33%	71	FGFR1	1	0,39%	2,33%
72	PDCD1LG2	1	0,39%	2,33%	72	FGFR3	1	0,39%	2,33%
73	PDGFRA	1	0,39%	2,33%	73	FUBP1	1	0,39%	2,33%
74	PIK3CA	7	2,72%	16,28%	74	GATA4	1	0,39%	2,33%
75	PMS2	1	0,39%	2,33%	75	HGF	1	0,39%	2,33%
76	PRKCI	6	2,33%	13,95%	76	HSD3B1	1	0,39%	2,33%
77	PTEN	6	2,33%	13,95%	77	JAK2	1	0,39%	2,33%
78	RAD21	1	0,39%	2,33%	78	JUN	1	0,39%	2,33%
79	RAF1	1	0,39%	2,33%	79	KEL	1	0,39%	2,33%
80	RB1	4	1,56%	9,30%	80	MAPK1	1	0,39%	2,33%
81	RBM10	3	1,17%	6,98%	81	MTOR	1	0,39%	2,33%
82	RET	1	0,39%	2,33%	82	PAX5	1	0,39%	2,33%
83	RICTOR	1	0,39%	2,33%	83	PDCD1	1	0,39%	2,33%
84	SETD2	1	0,39%	2,33%	84	PDCD1LG2	1	0,39%	2,33%

85	SMAD4	1	0,39%	2,33%	85	PDGFRA	1	0,39%	2,33%
86	SMARCA4	2	0,78%	4,65%	86	PMS2	1	0,39%	2,33%
87	SMARCB1	1	0,39%	2,33%	87	RAD21	1	0,39%	2,33%
88	SOX2	5	1,95%	11,63%	88	RAF1	1	0,39%	2,33%
89	SPEN	3	1,17%	6,98%	89	RET	1	0,39%	2,33%
90	SPOP	1	0,39%	2,33%	90	RICTOR	1	0,39%	2,33%
91	STAG2	1	0,39%	2,33%	91	SETD2	1	0,39%	2,33%
92	STK11	8	3,11%	18,60%	92	SMAD4	1	0,39%	2,33%
93	TERC	5	1,95%	11,63%	93	SMARCB1	1	0,39%	2,33%
94	TERT	1	0,39%	2,33%	94	SPOP	1	0,39%	2,33%
95	TET2	2	0,78%	4,65%	95	STAG2	1	0,39%	2,33%
96	TP53	31	12,06%	72,09%	96	TERT	1	0,39%	2,33%
97	TSC2	1	0,39%	2,33%	97	TSC2	1	0,39%	2,33%
98	XRCC2	1	0,39%	2,33%	98	XRCC2	1	0,39%	2,33%
	Total	257	100,00%			Total	257	100,00%	

Total number of patients tested n=43. Total number of genetic abnormalities found n=257. Genes in red may represent clonal hematopoesis.

Table S4. Genetic abnormalities found at baseline in liquid biopsy (F1LCDx Blood)

	Gene, alphabetical	N muts	% genes (n=453)	% pts (n=99)		Gene, descending frequency	N muts	% genes (n=453)	% pts (n=99)
1	AKT2	1	0,22%	1,01%	1	TP53	68	15,01%	68,69%
2	ALK	3	0,66%	3,03%	2	DNMT3A	33	7,28%	33,33%
3	APC	5	1,10%	5,05%	3	KRAS	22	4,86%	22,22%
4	ARAF	2	0,44%	2,02%	4	TET2	19	4,19%	19,19%
5	ARFRP1	1	0,22%	1,01%	5	ASXL1	16	3,53%	16,16%
6	ARID1A	4	0,88%	4,04%	6	STK11	14	3,09%	14,14%
7	ASXL1	16	3,53%	16,16%	7	EGFR	13	2,87%	13,13%
8	ATM	9	1,99%	9,09%	8	NF1	13	2,87%	13,13%
9	AURKA	1	0,22%	1,01%	9	ATM	9	1,99%	9,09%
10	BARD1	1	0,22%	1,01%	10	CDKN2A/B	9	1,99%	9,09%
11	BCOR	6	1,32%	6,06%	11	CHEK2	9	1,99%	9,09%
12	BCORL1	2	0,44%	2,02%	12	KEAP1	9	1,99%	9,09%
13	BRAF	3	0,66%	3,03%	13	RB1	7	1,55%	7,07%
14	BRCA1	1	0,22%	1,01%	14	SF3B1	7	1,55%	7,07%
15	BRCA2	2	0,44%	2,02%	15	BCOR	6	1,32%	6,06%
16	CBL	6	1,32%	6,06%	16	CBL	6	1,32%	6,06%
17	CCND1	2	0,44%	2,02%	17	JAK2	6	1,32%	6,06%
18	CCNE1	1	0,22%	1,01%	18	KMT2D	6	1,32%	6,06%
19	CDH1	1	0,22%	1,01%	19	NOTCH1	6	1,32%	6,06%
20	CDK12	2	0,44%	2,02%	20	PIK3CA	6	1,32%	6,06%
21	CDK4	1	0,22%	1,01%	21	PTEN	6	1,32%	6,06%
22	CDKN21/b	1	0,22%	1,01%	22	APC	5	1,10%	5,05%
23	CDKN2A/B	9	1,99%	9,09%	23	ERBB2	5	1,10%	5,05%
24	CDKN2C	1	0,22%	1,01%	24	KDM6A	5	1,10%	5,05%
25	CHEK2	9	1,99%	9,09%	25	RBM10	5	1,10%	5,05%
26	CREBBP	3	0,66%	3,03%	26	ARID1A	4	0,88%	4,04%
27	CRKL	2	0,44%	2,02%	27	PTPN11	4	0,88%	4,04%
28	CTCF	2	0,44%	2,02%	28	ALK	3	0,66%	3,03%
29	CTNNA1	1	0,22%	1,01%	29	BRAF	3	0,66%	3,03%
30	CTNNB1	2	0,44%	2,02%	30	CREBBP	3	0,66%	3,03%
31	CUL3	3	0,66%	3,03%	31	CUL3	3	0,66%	3,03%
32	DNMT3A	33	7,28%	33,33%	32	EP300	3	0,66%	3,03%
33	EGFR	13	2,87%	13,13%	33	FANCL	3	0,66%	3,03%
34	EP300	3	0,66%	3,03%	34	GNAS	3	0,66%	3,03%
35	EPHA3	1	0,22%	1,01%	35	MYC	3	0,66%	3,03%
36	ERBB2	5	1,10%	5,05%	36	NFE2L2	3	0,66%	3,03%
37	EZH2	1	0,22%	1,01%	37	TERT	3	0,66%	3,03%
38	FANCA	1	0,22%	1,01%	38	ARAF	2	0,44%	2,02%
39	FANCC	1	0,22%	1,01%	39	BCORL1	2	0,44%	2,02%
40	FANCL	3	0,66%	3,03%	40	BRCA2	2	0,44%	2,02%

41	FBXW7	2	0,44%	2,02%	41	CCND1	2	0,44%	2,02%
42	FGF12	1	0,22%	1,01%	42	CDK12	2	0,44%	2,02%
43	FGF19	2	0,44%	2,02%	43	CRKL	2	0,44%	2,02%
44	FGF3	2	0,44%	2,02%	44	CTCF	2	0,44%	2,02%
45	FGF4	2	0,44%	2,02%	45	CTNNB1	2	0,44%	2,02%
46	FGFR1	2	0,44%	2,02%	46	FBXW7	2	0,44%	2,02%
47	FGFR3	2	0,44%	2,02%	47	FGF19	2	0,44%	2,02%
48	FUBP1	1	0,22%	1,01%	48	FGF3	2	0,44%	2,02%
49	GNAS	3	0,66%	3,03%	49	FGF4	2	0,44%	2,02%
50	HSD3B1	2	0,44%	2,02%	50	FGFR1	2	0,44%	2,02%
51	IDH1	1	0,22%	1,01%	51	FGFR3	2	0,44%	2,02%
52	IDH2	2	0,44%	2,02%	52	HSD3B1	2	0,44%	2,02%
53	IRF2	1	0,22%	1,01%	53	IDH2	2	0,44%	2,02%
54	JAK2	6	1,32%	6,06%	54	MDM2	2	0,44%	2,02%
55	KDM5C	1	0,22%	1,01%	55	NBN	2	0,44%	2,02%
56	KDM6A	5	1,10%	5,05%	56	NSD3 (WHSC1L1)	2	0,44%	2,02%
57	KEAP1	9	1,99%	9,09%	57	PALB2	2	0,44%	2,02%
58	KMT2D	6	1,32%	6,06%	58	PBRM1	2	0,44%	2,02%
59	KRAS	22	4,86%	22,22%	59	PDGFRA	2	0,44%	2,02%
60	MDM2	2	0,44%	2,02%	60	PIK3R1	2	0,44%	2,02%
61	MEN1	1	0,22%	1,01%	61	REL	2	0,44%	2,02%
62	MET	1	0,22%	1,01%	62	SETD2	2	0,44%	2,02%
63	MLL2	1	0,22%	1,01%	63	SMARCA4	2	0,44%	2,02%
64	MSH6	1	0,22%	1,01%	64	SOX2	2	0,44%	2,02%
65	MTOR	1	0,22%	1,01%	65	STAG2	2	0,44%	2,02%
66	MYC	3	0,66%	3,03%	66	TSC2	2	0,44%	2,02%
67	MYCL1	1	0,22%	1,01%	67	AKT2	1	0,22%	1,01%
68	NBN	2	0,44%	2,02%	68	ARFRP1	1	0,22%	1,01%
69	NF1	13	2,87%	13,13%	69	AURKA	1	0,22%	1,01%
70	NFE2L2	3	0,66%	3,03%	70	BARD1	1	0,22%	1,01%
71	NKX2-1	1	0,22%	1,01%	71	BRCA1	1	0,22%	1,01%
72	NOTCH1	6	1,32%	6,06%	72	CCNE1	1	0,22%	1,01%
73	NOTCH2	1	0,22%	1,01%	73	CDH1	1	0,22%	1,01%
74	NRAS	1	0,22%	1,01%	74	CDK4	1	0,22%	1,01%
75	NSD3 (WHSC1L1)	2	0,44%	2,02%	75	CDKN21/b	1	0,22%	1,01%
76	PALB2	2	0,44%	2,02%	76	CDKN2C	1	0,22%	1,01%
77	PAX5	1	0,22%	1,01%	77	CTNNA1	1	0,22%	1,01%
78	PBRM1	2	0,44%	2,02%	78	EPHA3	1	0,22%	1,01%
79	PDGFRA	2	0,44%	2,02%	79	EZH2	1	0,22%	1,01%
80	PIK3CA	6	1,32%	6,06%	80	FANCA	1	0,22%	1,01%
81	PIK3R1	2	0,44%	2,02%	81	FANCC	1	0,22%	1,01%
82	PMS2	1	0,22%	1,01%	82	FGF12	1	0,22%	1,01%
83	PRDM1	1	0,22%	1,01%	83	FUBP1	1	0,22%	1,01%
84	PRKCI	1	0,22%	1,01%	84	IDH1	1	0,22%	1,01%

85	PTEN	6	1,32%	6,06%	85	IRF2	1	0,22%	1,01%
86	PTPN11	4	0,88%	4,04%	86	KDM5C	1	0,22%	1,01%
87	QKI	1	0,22%	1,01%	87	MEN1	1	0,22%	1,01%
88	RAD21	1	0,22%	1,01%	88	MET	1	0,22%	1,01%
89	RAD51C	1	0,22%	1,01%	89	MLL2	1	0,22%	1,01%
90	RAD54L	1	0,22%	1,01%	90	MSH6	1	0,22%	1,01%
91	RAF1	1	0,22%	1,01%	91	MTOR	1	0,22%	1,01%
92	RB1	7	1,55%	7,07%	92	MYCL1	1	0,22%	1,01%
93	RBM10	5	1,10%	5,05%	93	NKX2-1	1	0,22%	1,01%
94	REL	2	0,44%	2,02%	94	NOTCH2	1	0,22%	1,01%
95	RNF43	1	0,22%	1,01%	95	NRAS	1	0,22%	1,01%
96	SETD2	2	0,44%	2,02%	96	PAX5	1	0,22%	1,01%
97	SF3B1	7	1,55%	7,07%	97	PMS2	1	0,22%	1,01%
98	SMAD4	1	0,22%	1,01%	98	PRDM1	1	0,22%	1,01%
99	SMARCA4	2	0,44%	2,02%	99	PRKCI	1	0,22%	1,01%
100	SOX2	2	0,44%	2,02%	100	QKI	1	0,22%	1,01%
101	SPEN	1	0,22%	1,01%	101	RAD21	1	0,22%	1,01%
102	SPOP	1	0,22%	1,01%	102	RAD51C	1	0,22%	1,01%
103	STAG2	2	0,44%	2,02%	103	RAD54L	1	0,22%	1,01%
104	STAT3	1	0,22%	1,01%	104	RAF1	1	0,22%	1,01%
105	STK11	14	3,09%	14,14%	105	RNF43	1	0,22%	1,01%
106	TEK	1	0,22%	1,01%	106	SMAD4	1	0,22%	1,01%
107	TERT	3	0,66%	3,03%	107	SPEN	1	0,22%	1,01%
108	TET2	19	4,19%	19,19%	108	SPOP	1	0,22%	1,01%
109	TP53	68	15,01%	68,69%	109	STAT3	1	0,22%	1,01%
110	TSC2	2	0,44%	2,02%	110	TEK	1	0,22%	1,01%
111	U2AF1	1	0,22%	1,01%	111	U2AF1	1	0,22%	1,01%
112	XRCC2	1	0,22%	1,01%	112	XRCC2	1	0,22%	1,01%
113	ZNF703	1	0,22%	1,01%	113	ZNF703	1	0,22%	1,01%
114	No Mut (3 pts)	0	0,00%	0,00%	114	No Mut (3 pts)	0	0,00%	0,00%
	Total	453	100,00%			Total	453	100,00%	

Total number of patients tested n=99. Total number of genetic abnormalities found n=453. Genes in red may represent clonal hematopoesis.

Table S5. Genetic abnormalities found at progression with liquid biopsy (F1LCDx Blood).

	Gene, alphabetical	N muts	% genes (n=146)	% pts (n=34)		Gene, descending frequency	N muts	% genes (n=146)	% pts (n=34)
1	ACVR1B	1	0,68%	2,94%	1	TP53	24	16,44%	70,59%
2	APC	2	1,37%	5,88%	2	KRAS	12	8,22%	35,29%
3	ARID1A	1	0,68%	2,94%	3	DNMT3A	11	7,53%	32,35%
4	ASXL1	7	4,79%	20,59%	4	ASXL1	7	4,79%	20,59%
5	ATM	5	3,42%	14,71%	5	STK11	7	4,79%	20,59%
6	BARD1	1	0,68%	2,94%	6	KEAP1	6	4,11%	17,65%
7	BRAF	2	1,37%	5,88%	7	ATM	5	3,42%	14,71%
8	BRCA2	2	1,37%	5,88%	8	CHEK2	5	3,42%	14,71%
9	CBL	3	2,05%	8,82%	9	NF1	5	3,42%	14,71%
10	CDKN2A/B	4	2,74%	11,76%	10	CDKN2A/B	4	2,74%	11,76%
11	CDKN2C	1	0,68%	2,94%	11	TET2	4	2,74%	11,76%
12	CHEK2	5	3,42%	14,71%	12	CBL	3	2,05%	8,82%
13	CREBBP	1	0,68%	2,94%	13	EGFR	3	2,05%	8,82%
14	CRKL	1	0,68%	2,94%	14	JAK2	3	2,05%	8,82%
15	DNMT3A	11	7,53%	32,35%	15	APC	2	1,37%	5,88%
16	EGFR	3	2,05%	8,82%	16	BRAF	2	1,37%	5,88%
17	EPHA3	1	0,68%	2,94%	17	BRCA2	2	1,37%	5,88%
18	ERBB2	1	0,68%	2,94%	18	KMT2D	2	1,37%	5,88%
19	EZH2	1	0,68%	2,94%	19	MEN1	2	1,37%	5,88%
20	FANCG	1	0,68%	2,94%	20	MYC	2	1,37%	5,88%
21	FBXW7	1	0,68%	2,94%	21	NOTCH1	2	1,37%	5,88%
22	FGFR2	1	0,68%	2,94%	22	RAD21	2	1,37%	5,88%
23	FGFR2B	1	0,68%	2,94%	23	RBM10	2	1,37%	5,88%
24	GNAS	1	0,68%	2,94%	24	ACVR1B	1	0,68%	2,94%
25	IRF2	1	0,68%	2,94%	25	ARID1A	1	0,68%	2,94%
26	JAK2	3	2,05%	8,82%	26	BARD1	1	0,68%	2,94%
27	KEAP1	6	4,11%	17,65%	27	CDKN2C	1	0,68%	2,94%
28	KMT2D	2	1,37%	5,88%	28	CREBBP	1	0,68%	2,94%
29	KRAS	12	8,22%	35,29%	29	CRKL	1	0,68%	2,94%
30	MAP2K1 (MEK)	1	0,68%	2,94%	30	EPHA3	1	0,68%	2,94%
31	MEN1	2	1,37%	5,88%	31	ERBB2	1	0,68%	2,94%
32	MLL2	1	0,68%	2,94%	32	EZH2	1	0,68%	2,94%
33	MYC	2	1,37%	5,88%	33	FANCG	1	0,68%	2,94%
34	NF1	5	3,42%	14,71%	34	FBXW7	1	0,68%	2,94%
35	NFKBIA	1	0,68%	2,94%	35	FGFR2	1	0,68%	2,94%
36	NKX2-1	1	0,68%	2,94%	36	FGFR2B	1	0,68%	2,94%
37	NOTCH1	2	1,37%	5,88%	37	GNAS	1	0,68%	2,94%
38	NTRK1	1	0,68%	2,94%	38	IRF2	1	0,68%	2,94%
39	PDGFRB	1	0,68%	2,94%	39	MAP2K1 (MEK)	1	0,68%	2,94%
40	PIK3CA	1	0,68%	2,94%	40	MLL2	1	0,68%	2,94%

41	PPP2R2A	1	0,68%	2,94%	41	NFKBIA	1	0,68%	2,94%
42	PRKCI	1	0,68%	2,94%	42	NKX2-1	1	0,68%	2,94%
43	PTEN	1	0,68%	2,94%	43	NTRK1	1	0,68%	2,94%
44	PTPRO	1	0,68%	2,94%	44	PDGFRB	1	0,68%	2,94%
45	RAD21	2	1,37%	5,88%	45	PIK3CA	1	0,68%	2,94%
46	RBM10	2	1,37%	5,88%	46	PPP2R2A	1	0,68%	2,94%
47	SETD2	1	0,68%	2,94%	47	PRKCI	1	0,68%	2,94%
48	SF3B1	1	0,68%	2,94%	48	PTEN	1	0,68%	2,94%
49	STK11	7	4,79%	20,59%	49	PTPRO	1	0,68%	2,94%
50	TERC	1	0,68%	2,94%	50	SETD2	1	0,68%	2,94%
51	TET2	4	2,74%	11,76%	51	SF3B1	1	0,68%	2,94%
52	TP53	24	16,44%	70,59%	52	TERC	1	0,68%	2,94%
53	No mut (1 pt)	0	0,00%	0,00%	53	No mut (1 pt)	0	0,00%	0,00%
	Total	146	100,00%			Total	146	100,00%	

Total number of patients tested n=34. Total number of genetic abnormalities found n=146. Genes in red may represent clonal hematopoesis.

Table S6. Genetic abnormalities found at baseline with tissue or liquid biopsy (F1CDX tissue, F1LCDx Blood) in non-squamous NSCLC

	Gene, alphabetical	N muts	% genes (n=316)	% pts (n=66)
1	AKT1	1	0,32%	1,52%
2	AKT2	1	0,32%	1,52%
3	ALK	2	0,63%	3,03%
4	APC	3	0,95%	4,55%
5	ARAF	2	0,63%	3,03%
6	ARFRP1	1	0,32%	1,52%
7	ARID1A	2	0,63%	3,03%
8	ASXL1	7	2,22%	10,61%
9	ATM	7	2,22%	10,61%
10	AURKA	1	0,32%	1,52%
11	BARD1	1	0,32%	1,52%
12	BCOR	4	1,27%	6,06%
13	BCORL1	1	0,32%	1,52%
14	BRAF	3	0,95%	4,55%
15	CARD11	1	0,32%	1,52%
16	CBL	3	0,95%	4,55%
17	CCNE	1	0,32%	1,52%
18	CCNE1	2	0,63%	3,03%
19	CDK12	2	0,63%	3,03%
20	CDK4	2	0,63%	3,03%
21	CDK6	1	0,32%	1,52%
22	CDKN1B	1	0,32%	1,52%
23	CDKN21/b	1	0,32%	1,52%
24	CDKN2A/B	7	2,22%	10,61%
25	CDKN2C	1	0,32%	1,52%
26	CHEK2	2	0,63%	3,03%
27	CIC	1	0,32%	1,52%
28	CREBBP	1	0,32%	1,52%
29	CTCF	1	0,32%	1,52%
30	CTNNA1	1	0,32%	1,52%
31	CTNNB1	2	0,63%	3,03%
32	CUL3	2	0,63%	3,03%
33	DNMT3A	23	7,28%	34,85%
34	EGFR	14	4,43%	21,21%
35	EP300	1	0,32%	1,52%
36	ERBB2	4	1,27%	6,06%
37	EZH2	1	0,32%	1,52%
38	FANCL	1	0,32%	1,52%
39	FGF10	1	0,32%	1,52%
40	FGFR1	1	0,32%	1,52%

	Gene, descending frequency	N muts	% genes (n=316)	% pts (n=66)
1	TP53	41	12,97%	62,12%
2	DNMT3A	23	7,28%	34,85%
3	KRAS	22	6,96%	33,33%
4	STK11	15	4,75%	22,73%
5	EGFR	14	4,43%	21,21%
6	TET2	10	3,16%	15,15%
7	ASXL1	7	2,22%	10,61%
8	ATM	7	2,22%	10,61%
9	CDKN2A/B	7	2,22%	10,61%
10	KEAP1	7	2,22%	10,61%
11	NF1	7	2,22%	10,61%
12	RB1	6	1,90%	9,09%
13	JAK2	5	1,58%	7,58%
14	MTAP	5	1,58%	7,58%
15	BCOR	4	1,27%	6,06%
16	ERBB2	4	1,27%	6,06%
17	RBM10	4	1,27%	6,06%
18	SF3B1	4	1,27%	6,06%
19	APC	3	0,95%	4,55%
20	BRAF	3	0,95%	4,55%
21	CBL	3	0,95%	4,55%
22	KDM6A	3	0,95%	4,55%
23	KMT2D	3	0,95%	4,55%
24	PIK3CA	3	0,95%	4,55%
25	SETD2	3	0,95%	4,55%
26	SMARCA4	3	0,95%	4,55%
27	ALK	2	0,63%	3,03%
28	ARAF	2	0,63%	3,03%
29	ARID1A	2	0,63%	3,03%
30	CCNE1	2	0,63%	3,03%
31	CDK12	2	0,63%	3,03%
32	CDK4	2	0,63%	3,03%
33	CHEK2	2	0,63%	3,03%
34	CTNNB1	2	0,63%	3,03%
35	CUL3	2	0,63%	3,03%
36	GNAS	2	0,63%	3,03%
37	HSD3B1	2	0,63%	3,03%
38	MAP2K4	2	0,63%	3,03%
39	MYC	2	0,63%	3,03%
40	NF2	2	0,63%	3,03%

41	FUBP1	1	0,32%	1,52%
42	GNAS	2	0,63%	3,03%
43	HGF	1	0,32%	1,52%
44	HSD3B1	2	0,63%	3,03%
45	IDH2	1	0,32%	1,52%
46	IRF2	1	0,32%	1,52%
47	JAK2	5	1,58%	7,58%
48	KDM5C	1	0,32%	1,52%
49	KDM6A	3	0,95%	4,55%
50	KEAP1	7	2,22%	10,61%
51	KMT2D	3	0,95%	4,55%
52	KRAS	22	6,96%	33,33%
53	MAP2K4	2	0,63%	3,03%
54	MDM2	1	0,32%	1,52%
55	MEN1	1	0,32%	1,52%
56	MET	1	0,32%	1,52%
57	MSH6	1	0,32%	1,52%
58	MTAP	5	1,58%	7,58%
59	MYC	2	0,63%	3,03%
60	MYCL1	1	0,32%	1,52%
61	NF1	7	2,22%	10,61%
62	NF2	2	0,63%	3,03%
63	NFKBIA	1	0,32%	1,52%
64	NKX2-1	2	0,63%	3,03%
65	NOTCH1	2	0,63%	3,03%
66	NRAS	1	0,32%	1,52%
67	NSD3 (WHSC1L1)	2	0,63%	3,03%
68	PALB2	1	0,32%	1,52%
69	PAX5	1	0,32%	1,52%
70	PBRM1	2	0,63%	3,03%
71	PDCD1	1	0,32%	1,52%
72	PDCD1LG2	1	0,32%	1,52%
73	PDGFRA	2	0,63%	3,03%
74	PIK3CA	3	0,95%	4,55%
75	PIK3R1	2	0,63%	3,03%
76	PMS2	1	0,32%	1,52%
77	PRDM1	1	0,32%	1,52%
78	PTEN	1	0,32%	1,52%
79	PTPN11	2	0,63%	3,03%
80	RAD21	1	0,32%	1,52%
81	RAD54L	1	0,32%	1,52%
82	RAF1	2	0,63%	3,03%
83	RB1	6	1,90%	9,09%
84	RBM10	4	1,27%	6,06%

41	NKX2-1	2	0,63%	3,03%
42	NOTCH1	2	0,63%	3,03%
43	NSD3 (WHSC1L1)	2	0,63%	3,03%
44	PBRM1	2	0,63%	3,03%
45	PDGFRA	2	0,63%	3,03%
46	PIK3R1	2	0,63%	3,03%
47	PTPN11	2	0,63%	3,03%
48	RAF1	2	0,63%	3,03%
49	SPEN	2	0,63%	3,03%
50	STAG2	2	0,63%	3,03%
51	TERT	2	0,63%	3,03%
52	TSC2	2	0,63%	3,03%
53	AKT1	1	0,32%	1,52%
54	AKT2	1	0,32%	1,52%
55	ARFRP1	1	0,32%	1,52%
56	AURKA	1	0,32%	1,52%
57	BARD1	1	0,32%	1,52%
58	BCORL1	1	0,32%	1,52%
59	CARD11	1	0,32%	1,52%
60	CCNE	1	0,32%	1,52%
61	CDK6	1	0,32%	1,52%
62	CDKN1B	1	0,32%	1,52%
63	CDKN21/b	1	0,32%	1,52%
64	CDKN2C	1	0,32%	1,52%
65	CIC	1	0,32%	1,52%
66	CREBBP	1	0,32%	1,52%
67	CTCF	1	0,32%	1,52%
68	CTNNA1	1	0,32%	1,52%
69	EP300	1	0,32%	1,52%
70	EZH2	1	0,32%	1,52%
71	FANCL	1	0,32%	1,52%
72	FGF10	1	0,32%	1,52%
73	FGFR1	1	0,32%	1,52%
74	FUBP1	1	0,32%	1,52%
75	HGF	1	0,32%	1,52%
76	IDH2	1	0,32%	1,52%
77	IRF2	1	0,32%	1,52%
78	KDM5C	1	0,32%	1,52%
79	MDM2	1	0,32%	1,52%
80	MEN1	1	0,32%	1,52%
81	MET	1	0,32%	1,52%
82	MSH6	1	0,32%	1,52%
83	MYCL1	1	0,32%	1,52%
84	NFKBIA	1	0,32%	1,52%

85	REL	1	0,32%	1,52%
86	RNF43	1	0,32%	1,52%
87	SETD2	3	0,95%	4,55%
88	SF3B1	4	1,27%	6,06%
89	SMAD4	1	0,32%	1,52%
90	SMARCA4	3	0,95%	4,55%
91	SPEN	2	0,63%	3,03%
92	STAG2	2	0,63%	3,03%
93	STK11	15	4,75%	22,73%
94	TERT	2	0,63%	3,03%
95	TET2	10	3,16%	15,15%
96	TP53	41	12,97%	62,12%
97	TSC2	2	0,63%	3,03%
98	U2AF1	1	0,32%	1,52%
99	XRCC2	1	0,32%	1,52%
100	ZNF703	1	0,32%	1,52%
101	No muts (2 pts)	0	0,00%	0,00%
	Total	316	100,00%	

85	NRAS	1	0,32%	1,52%
86	PALB2	1	0,32%	1,52%
87	PAX5	1	0,32%	1,52%
88	PDCD1	1	0,32%	1,52%
89	PDCD1LG2	1	0,32%	1,52%
90	PMS2	1	0,32%	1,52%
91	PRDM1	1	0,32%	1,52%
92	PTEN	1	0,32%	1,52%
93	RAD21	1	0,32%	1,52%
94	RAD54L	1	0,32%	1,52%
95	REL	1	0,32%	1,52%
96	RNF43	1	0,32%	1,52%
97	SMAD4	1	0,32%	1,52%
98	U2AF1	1	0,32%	1,52%
99	XRCC2	1	0,32%	1,52%
100	ZNF703	1	0,32%	1,52%
101	No muts (2 pts)	0	0,00%	0,00%
	Total	316	100,00%	

Total number of patients tested N=66. Total number of genetic abnormalities found n=316. Of the 22 KRAS mutated patients, 7 had a G12C mutation. Genes in red may represent clonal hematopoesis.

Table S7. Genetic abnormalities found at baseline with tissue or liquid biopsy (F1CDX tissue, F1LCDx Blood) in squamous NSCLC

	Gene, alphabetical	N muts	% genes (n=271)	% pts (n=38)		Gene, descending frequency	N muts	% gene (n=271)	% pts (n=38)
1	ALK	1	0,37%	2,63%	1	TP53	31	11,44%	81,58%
2	APC	2	0,74%	5,26%	2	CDKN2A/B	13	4,80%	34,21%
3	ARID1A	4	1,48%	10,53%	3	DNMT3A	10	3,69%	26,32%
4	ASXL1	9	3,32%	23,68%	4	ASXL1	9	3,32%	23,68%
5	ATM	2	0,74%	5,26%	5	NF1	9	3,32%	23,68%
6	AURKB	1	0,37%	2,63%	6	TET2	9	3,32%	23,68%
7	BCL2L1	1	0,37%	2,63%	7	CHEK2	7	2,58%	18,42%
8	BCOR	2	0,74%	5,26%	8	PIK3CA	7	2,58%	18,42%
9	BCORL1	1	0,37%	2,63%	9	PTEN	7	2,58%	18,42%
10	BRAF	2	0,74%	5,26%	10	PRKCI	6	2,21%	15,79%
11	BRCA1	1	0,37%	2,63%	11	KEAP1	5	1,85%	13,16%
12	BRCA2	2	0,74%	5,26%	12	MTAP	5	1,85%	13,16%
13	CBL	3	1,11%	7,89%	13	MYC	5	1,85%	13,16%
14	CCND1	3	1,11%	7,89%	14	NOTCH1	5	1,85%	13,16%
15	CDH1	1	0,37%	2,63%	15	SOX2	5	1,85%	13,16%
16	CDK4	1	0,37%	2,63%	16	TERC	5	1,85%	13,16%
17	CDKN2A/B	13	4,80%	34,21%	17	ARID1A	4	1,48%	10,53%
18	CHEK2	7	2,58%	18,42%	18	KRAS	4	1,48%	10,53%
19	CREBBP	2	0,74%	5,26%	19	CBL	3	1,11%	7,89%
20	CRKL	2	0,74%	5,26%	20	CCND1	3	1,11%	7,89%
21	CTCF	1	0,37%	2,63%	21	ERBB2	3	1,11%	7,89%
22	CUL3	1	0,37%	2,63%	22	FGF12	3	1,11%	7,89%
23	DKN2A/B	1	0,37%	2,63%	23	FGF19	3	1,11%	7,89%
24	DNMT3A	10	3,69%	26,32%	24	FGF3	3	1,11%	7,89%
25	EGFR	2	0,74%	5,26%	25	KDM6A	3	1,11%	7,89%
26	EP300	2	0,74%	5,26%	26	KMT2D	3	1,11%	7,89%
27	EPHA3	1	0,37%	2,63%	27	NFE2L2	3	1,11%	7,89%
28	ERBB2	3	1,11%	7,89%	28	RB1	3	1,11%	7,89%
29	FANCA	2	0,74%	5,26%	29	SF3B1	3	1,11%	7,89%
30	FANCC	1	0,37%	2,63%	30	STK11	3	1,11%	7,89%
31	FANCL	2	0,74%	5,26%	31	APC	2	0,74%	5,26%
32	FBXW7	2	0,74%	5,26%	32	ATM	2	0,74%	5,26%
33	FGF10	1	0,37%	2,63%	33	BCOR	2	0,74%	5,26%
34	FGF12	3	1,11%	7,89%	34	BRAF	2	0,74%	5,26%
35	FGF19	3	1,11%	7,89%	35	BRCA2	2	0,74%	5,26%
36	FGF3	3	1,11%	7,89%	36	CREBBP	2	0,74%	5,26%
37	FGF4	2	0,74%	5,26%	37	CRKL	2	0,74%	5,26%
38	FGFR1	2	0,74%	5,26%	38	EGFR	2	0,74%	5,26%
39	FGFR3	2	0,74%	5,26%	39	EP300	2	0,74%	5,26%

40	GATA4	1	0,37%	2,63%
41	GNAS	1	0,37%	2,63%
42	IDH1	1	0,37%	2,63%
43	IDH2	1	0,37%	2,63%
44	JAK2	2	0,74%	5,26%
45	JUN	1	0,37%	2,63%
46	KDM5A	2	0,74%	5,26%
47	KDM6A	3	1,11%	7,89%
48	KEAP1	5	1,85%	13,16%
49	KEL	1	0,37%	2,63%
50	KMT2D	3	1,11%	7,89%
51	KRAS	4	1,48%	10,53%
52	MAPK1	1	0,37%	2,63%
53	MDM2	2	0,74%	5,26%
54	MET	1	0,37%	2,63%
55	MLL2	1	0,37%	2,63%
56	MTAP	5	1,85%	13,16%
57	MTOR	1	0,37%	2,63%
58	MYC	5	1,85%	13,16%
59	MYCL1	2	0,74%	5,26%
60	NBN	2	0,74%	5,26%
61	NF1	9	3,32%	23,68%
62	NFE2L2	3	1,11%	7,89%
63	NFKBIA	1	0,37%	2,63%
64	NKX2-1	2	0,74%	5,26%
65	NOTCH1	5	1,85%	13,16%
66	NOTCH2	2	0,74%	5,26%
67	NSD3 (WHSC1L1)	2	0,74%	5,26%
68	PALB2	1	0,37%	2,63%
69	PIK3CA	7	2,58%	18,42%
70	PRKCI	6	2,21%	15,79%
71	PTEN	7	2,58%	18,42%
72	PTPN11	2	0,74%	5,26%
73	QKI	1	0,37%	2,63%
74	RAD21	1	0,37%	2,63%
75	RAD51C	1	0,37%	2,63%
76	RB1	3	1,11%	7,89%
77	RBM10	2	0,74%	5,26%
78	REL	1	0,37%	2,63%
79	RET	1	0,37%	2,63%
80	RICTOR	1	0,37%	2,63%
81	SF3B1	3	1,11%	7,89%
82	SMAD4	1	0,37%	2,63%
83	SMARCB1	1	0,37%	2,63%

40	FANCA	2	0,74%	5,26%
41	FANCL	2	0,74%	5,26%
42	FBXW7	2	0,74%	5,26%
43	FGF4	2	0,74%	5,26%
44	FGFR1	2	0,74%	5,26%
45	FGFR3	2	0,74%	5,26%
46	JAK2	2	0,74%	5,26%
47	KDM5A	2	0,74%	5,26%
48	MDM2	2	0,74%	5,26%
49	MYCL1	2	0,74%	5,26%
50	NBN	2	0,74%	5,26%
51	NKX2-1	2	0,74%	5,26%
52	NOTCH2	2	0,74%	5,26%
53	NSD3 (WHSC1L1)	2	0,74%	5,26%
54	PTPN11	2	0,74%	5,26%
55	RBM10	2	0,74%	5,26%
56	ALK	1	0,37%	2,63%
57	AURKB	1	0,37%	2,63%
58	BCL2L1	1	0,37%	2,63%
59	BCORL1	1	0,37%	2,63%
60	BRCA1	1	0,37%	2,63%
61	CDH1	1	0,37%	2,63%
62	CDK4	1	0,37%	2,63%
63	CTCF	1	0,37%	2,63%
64	CUL3	1	0,37%	2,63%
65	DKN2A/B	1	0,37%	2,63%
66	EPHA3	1	0,37%	2,63%
67	FANCC	1	0,37%	2,63%
68	FGF10	1	0,37%	2,63%
69	GATA4	1	0,37%	2,63%
70	GNAS	1	0,37%	2,63%
71	IDH1	1	0,37%	2,63%
72	IDH2	1	0,37%	2,63%
73	JUN	1	0,37%	2,63%
74	KEL	1	0,37%	2,63%
75	MAPK1	1	0,37%	2,63%
76	MET	1	0,37%	2,63%
77	MLL2	1	0,37%	2,63%
78	MTOR	1	0,37%	2,63%
79	NFKBIA	1	0,37%	2,63%
80	PALB2	1	0,37%	2,63%
81	QKI	1	0,37%	2,63%
82	RAD21	1	0,37%	2,63%
83	RAD51C	1	0,37%	2,63%

84	SOX2	5	1,85%	13,16%
85	SPEN	1	0,37%	2,63%
86	SPOP	1	0,37%	2,63%
87	STAG2	1	0,37%	2,63%
88	STAT3	1	0,37%	2,63%
89	STK11	3	1,11%	7,89%
90	TEK	1	0,37%	2,63%
91	TERC	5	1,85%	13,16%
92	TERT	1	0,37%	2,63%
93	TET2	9	3,32%	23,68%
94	TP53	31	11,44%	81,58%
95	No muts (1 pt)	0	0,00%	0,00%
	Total	271	100,00%	

84	REL	1	0,37%	2,63%
85	RET	1	0,37%	2,63%
86	RICTOR	1	0,37%	2,63%
87	SMAD4	1	0,37%	2,63%
88	SMARCB1	1	0,37%	2,63%
89	SPEN	1	0,37%	2,63%
90	SPOP	1	0,37%	2,63%
91	STAG2	1	0,37%	2,63%
92	STAT3	1	0,37%	2,63%
93	TEK	1	0,37%	2,63%
94	TERT	1	0,37%	2,63%
95	No muts (1 pt)	0	0,00%	0,00%
	Total	271	100,00%	

Total number of patients tested N=38. Total number of genetic abnormalities found n=271. Genes in red may represent clonal hematopoesis.

Table S8. Genetic abnormalities found at baseline with tissue or liquid biopsy (F1CDX tissue, F1LCDx Blood) in active and past smokers

	Gene, alphabetical	N muts	% genes (n=471)	% pts (n=78)		Gene, descending frequency	N muts	% genes (n=472)	% pts (n=78)
1	AKT2	1	0,21%	1,28%	1	TP53	58	12,31%	74,36%
2	ALK	2	0,42%	2,56%	2	DNMT3A	23	4,88%	29,49%
3	APC	4	0,85%	5,13%	3	KRAS	23	4,88%	29,49%
4	ARAF	1	0,21%	1,28%	4	STK11	17	3,61%	21,79%
5	ARFRP1	1	0,21%	1,28%	5	ASXL1	16	3,40%	20,51%
6	ARID1A	5	1,06%	6,41%	6	TET2	15	3,18%	19,23%
7	ASXL1	16	3,40%	20,51%	7	CDKN2A/B	14	2,97%	17,95%
8	ATM	8	1,70%	10,26%	8	NF1	12	2,55%	15,38%
9	AURKA	1	0,21%	1,28%	9	KEAP1	10	2,12%	12,82%
10	AURKB	1	0,21%	1,28%	10	PIK3CA	9	1,91%	11,54%
11	BCL2L1	1	0,21%	1,28%	11	RB1	9	1,91%	11,54%
12	BCOR	5	1,06%	6,41%	12	ATM	8	1,70%	10,26%
13	BCORL1	2	0,42%	2,56%	13	CHEK2	8	1,70%	10,26%
14	BRAF	3	0,64%	3,85%	14	MTAP	7	1,49%	8,97%
15	BRCA1	1	0,21%	1,28%	15	NOTCH1	7	1,49%	8,97%
16	BRCA2	2	0,42%	2,56%	16	PTEN	7	1,49%	8,97%
17	CARD11	1	0,21%	1,28%	17	SF3B1	7	1,49%	8,97%
18	CBL	3	0,64%	3,85%	18	ERBB2	6	1,27%	7,69%
19	CCND1	3	0,64%	3,85%	19	KDM6A	6	1,27%	7,69%
20	CCNE	1	0,21%	1,28%	20	KMT2D	6	1,27%	7,69%
21	CCNE1	2	0,42%	2,56%	21	MYC	6	1,27%	7,69%
22	CDH1	1	0,21%	1,28%	22	ARID1A	5	1,06%	6,41%
23	CDK12	2	0,42%	2,56%	23	BCOR	5	1,06%	6,41%
24	CDK4	1	0,21%	1,28%	24	JAK2	5	1,06%	6,41%
25	CDKN1B	1	0,21%	1,28%	25	PRKCI	5	1,06%	6,41%
26	CDKN21/b	1	0,21%	1,28%	26	RBM10	5	1,06%	6,41%
27	CDKN2A/B	14	2,97%	17,95%	27	SOX2	5	1,06%	6,41%
28	CDKN2C	1	0,21%	1,28%	28	APC	4	0,85%	5,13%
29	CHEK2	8	1,70%	10,26%	29	EGFR	4	0,85%	5,13%
30	CIC	1	0,21%	1,28%	30	NKX2-1	4	0,85%	5,13%
31	CREBBP	2	0,42%	2,56%	31	PTPN11	4	0,85%	5,13%
32	CRKL	2	0,42%	2,56%	32	TERC	4	0,85%	5,13%
33	CTCF	1	0,21%	1,28%	33	BRAF	3	0,64%	3,85%
34	CTNNA1	1	0,21%	1,28%	34	CBL	3	0,64%	3,85%
35	CTNNB1	1	0,21%	1,28%	35	CCND1	3	0,64%	3,85%
36	CUL3	2	0,42%	2,56%	36	EP300	3	0,64%	3,85%
37	DKN2A/B	1	0,21%	1,28%	37	FANCL	3	0,64%	3,85%
38	DNMT3A	23	4,88%	29,49%	38	FGF12	3	0,64%	3,85%
39	EGFR	4	0,85%	5,13%	39	FGF19	3	0,64%	3,85%

40	EP300	3	0,64%	3,85%
41	EPHA3	1	0,21%	1,28%
42	ERBB2	6	1,27%	7,69%
43	FANCA	2	0,42%	2,56%
44	FANCC	1	0,21%	1,28%
45	FANCL	3	0,64%	3,85%
46	FBXW7	2	0,42%	2,56%
47	FGF10	2	0,42%	2,56%
48	FGF12	3	0,64%	3,85%
49	FGF19	3	0,64%	3,85%
50	FGF3	3	0,64%	3,85%
51	FGF4	2	0,42%	2,56%
52	FGFR1	2	0,42%	2,56%
53	FGFR3	2	0,42%	2,56%
54	FUBP1	1	0,21%	1,28%
55	GATA4	1	0,21%	1,28%
56	GNAS	2	0,42%	2,56%
57	HSD3B1	2	0,42%	2,56%
58	IDH1	1	0,21%	1,28%
59	IDH2	1	0,21%	1,28%
60	IRF2	1	0,21%	1,28%
61	JAK2	5	1,06%	6,41%
62	JUN	1	0,21%	1,28%
63	KDM5A	2	0,42%	2,56%
64	KDM5C	1	0,21%	1,28%
65	KDM6A	6	1,27%	7,69%
66	KEAP1	10	2,12%	12,82%
67	KEL	1	0,21%	1,28%
68	KMT2D	6	1,27%	7,69%
69	KRAS	23	4,88%	29,49%
70	MAP2K4	1	0,21%	1,28%
71	MAPK1	1	0,21%	1,28%
72	MDM2	1	0,21%	1,28%
73	MEN1	1	0,21%	1,28%
74	MLL2	1	0,21%	1,28%
75	MSH6	1	0,21%	1,28%
76	MTAP	7	1,49%	8,97%
77	MTOR	1	0,21%	1,28%
78	MYC	6	1,27%	7,69%
79	MYCL1	3	0,64%	3,85%
80	NBN	2	0,42%	2,56%
81	NF1	12	2,55%	15,38%
82	NF2	1	0,21%	1,28%
83	NFE2L2	3	0,64%	3,85%

40	FGF3	3	0,64%	3,85%
41	MYCL1	3	0,64%	3,85%
42	NFE2L2	3	0,64%	3,85%
43	NSD3 (WHSC1L1)	3	0,64%	3,85%
44	SETD2	3	0,64%	3,85%
45	SPEN	3	0,64%	3,85%
46	ALK	2	0,42%	2,56%
47	BCORL1	2	0,42%	2,56%
48	BRCA2	2	0,42%	2,56%
49	CCNE1	2	0,42%	2,56%
50	CDK12	2	0,42%	2,56%
51	CREBBP	2	0,42%	2,56%
52	CRKL	2	0,42%	2,56%
53	CUL3	2	0,42%	2,56%
54	FANCA	2	0,42%	2,56%
55	FBXW7	2	0,42%	2,56%
56	FGF10	2	0,42%	2,56%
57	FGF4	2	0,42%	2,56%
58	FGFR1	2	0,42%	2,56%
59	FGFR3	2	0,42%	2,56%
60	GNAS	2	0,42%	2,56%
61	HSD3B1	2	0,42%	2,56%
62	KDM5A	2	0,42%	2,56%
63	NBN	2	0,42%	2,56%
64	NFKBIA	2	0,42%	2,56%
65	NOTCH2	2	0,42%	2,56%
66	PALB2	2	0,42%	2,56%
67	PIK3R1	2	0,42%	2,56%
68	REL	2	0,42%	2,56%
69	STAG2	2	0,42%	2,56%
70	TERT	2	0,42%	2,56%
71	TSC2	2	0,42%	2,56%
72	AKT2	1	0,21%	1,28%
73	ARAF	1	0,21%	1,28%
74	ARFRP1	1	0,21%	1,28%
75	AURKA	1	0,21%	1,28%
76	AURKB	1	0,21%	1,28%
77	BCL2L1	1	0,21%	1,28%
78	BRCA1	1	0,21%	1,28%
79	CARD11	1	0,21%	1,28%
80	CCNE	1	0,21%	1,28%
81	CDH1	1	0,21%	1,28%
82	CDK4	1	0,21%	1,28%
83	CDKN1B	1	0,21%	1,28%

84	NFKBIA	2	0,42%	2,56%	84	CDKN21/b	1	0,21%	1,28%
85	NKX2-1	4	0,85%	5,13%	85	CDKN2C	1	0,21%	1,28%
86	NOTCH1	7	1,49%	8,97%	86	CIC	1	0,21%	1,28%
87	NOTCH2	2	0,42%	2,56%	87	CTCF	1	0,21%	1,28%
88	NSD3 (WHSC1L1)	3	0,64%	3,85%	88	CTNNA1	1	0,21%	1,28%
89	PALB2	2	0,42%	2,56%	89	CTNNB1	1	0,21%	1,28%
90	PAX5	1	0,21%	1,28%	90	DKN2A/B	1	0,21%	1,28%
91	PBRM1	1	0,21%	1,28%	91	EPHA3	1	0,21%	1,28%
92	PDCD1	1	0,21%	1,28%	92	FANCC	1	0,21%	1,28%
93	PDCD1LG2	1	0,21%	1,28%	93	FUBP1	1	0,21%	1,28%
94	PDGFRA	1	0,21%	1,28%	94	GATA4	1	0,21%	1,28%
95	PIK3CA	9	1,91%	11,54%	95	IDH1	1	0,21%	1,28%
96	PIK3R1	2	0,42%	2,56%	96	IDH2	1	0,21%	1,28%
97	PMS2	1	0,21%	1,28%	97	IRF2	1	0,21%	1,28%
98	PRDM1	1	0,21%	1,28%	98	JUN	1	0,21%	1,28%
99	PRKCI	5	1,06%	6,41%	99	KDM5C	1	0,21%	1,28%
100	PTEN	7	1,49%	8,97%	100	KEL	1	0,21%	1,28%
101	PTPN11	4	0,85%	5,13%	101	MAP2K4	1	0,21%	1,28%
102	QKI	1	0,21%	1,28%	102	MAPK1	1	0,21%	1,28%
103	RAD21	1	0,21%	1,28%	103	MDM2	1	0,21%	1,28%
104	RAD51C	1	0,21%	1,28%	104	MEN1	1	0,21%	1,28%
105	RAD54L	1	0,21%	1,28%	105	MLL2	1	0,21%	1,28%
106	RB1	9	1,91%	11,54%	106	MSH6	1	0,21%	1,28%
107	RBM10	5	1,06%	6,41%	107	MTOR	1	0,21%	1,28%
108	REL	2	0,42%	2,56%	108	NF2	1	0,21%	1,28%
109	RET	1	0,21%	1,28%	109	PAX5	1	0,21%	1,28%
110	RICTOR	1	0,21%	1,28%	110	PBRM1	1	0,21%	1,28%
111	RNF43	1	0,21%	1,28%	111	PDCD1	1	0,21%	1,28%
112	SETD2	3	0,64%	3,85%	112	PDCD1LG2	1	0,21%	1,28%
113	SF3B1	7	1,49%	8,97%	113	PDGFRA	1	0,21%	1,28%
114	SMAD4	1	0,21%	1,28%	114	PMS2	1	0,21%	1,28%
115	SMARCB1	1	0,21%	1,28%	115	PRDM1	1	0,21%	1,28%
116	SOX2	5	1,06%	6,41%	116	QKI	1	0,21%	1,28%
117	SPEN	3	0,64%	3,85%	117	RAD21	1	0,21%	1,28%
118	STAG2	2	0,42%	2,56%	118	RAD51C	1	0,21%	1,28%
119	STAT3	1	0,21%	1,28%	119	RAD54L	1	0,21%	1,28%
120	STK11	17	3,61%	21,79%	120	RET	1	0,21%	1,28%
121	TEK	1	0,21%	1,28%	121	RICTOR	1	0,21%	1,28%
122	TERC	4	0,85%	5,13%	122	RNF43	1	0,21%	1,28%
123	TERT	2	0,42%	2,56%	123	SMAD4	1	0,21%	1,28%
124	TET2	15	3,18%	19,23%	124	SMARCB1	1	0,21%	1,28%
125	TP53	58	12,31%	74,36%	125	STAT3	1	0,21%	1,28%
126	TSC2	2	0,42%	2,56%	126	TEK	1	0,21%	1,28%
127	U2AF1	1	0,21%	1,28%	127	U2AF1	1	0,21%	1,28%

128	No mut (1 pt)	0	0,00%	0,00%	128	No muts (1 pt)	0	0,00%	0,00%
	Total	471	100,00%			Total	471	100,00%	

Total number of patients tested N=78. Total number of genetic abnormalities found n=471. Genes in red may represent clonal hematopoesis.

Table S9. Genetic abnormalities found at baseline with tissue and/ or liquid biopsy (F1CDX tissue and/ or F1LCDx Blood) in never-smokers.

	Gene, alphabetical	N muts	% genes (n=116)	% pts (n=26)		Gene, descending frequency	N muts	% genes (n=116)	% pts (n=26)
1	AKT1	1	0,86%	3,85%	1	TP53	14	12,07%	53,85%
2	ALK	1	0,86%	3,85%	2	EGFR	12	10,34%	46,15%
3	APC	1	0,86%	3,85%	3	DNMT3A	10	8,62%	38,46%
4	ARAF	1	0,86%	3,85%	4	CDKN2A/B	6	5,17%	23,08%
5	ARID1A	1	0,86%	3,85%	5	NF1	4	3,45%	15,38%
6	ATM	1	0,86%	3,85%	6	TET2	4	3,45%	15,38%
7	BARD1	1	0,86%	3,85%	7	CBL	3	2,59%	11,54%
8	BCOR	1	0,86%	3,85%	8	KRAS	3	2,59%	11,54%
9	BRAF	2	1,72%	7,69%	9	MTAP	3	2,59%	11,54%
10	CBL	3	2,59%	11,54%	10	SMARCA4	3	2,59%	11,54%
11	CDK4	2	1,72%	7,69%	11	BRAF	2	1,72%	7,69%
12	CDK6	1	0,86%	3,85%	12	CDK4	2	1,72%	7,69%
13	CDKN2A/B	6	5,17%	23,08%	13	JAK2	2	1,72%	7,69%
14	CHEK2	1	0,86%	3,85%	14	KEAP1	2	1,72%	7,69%
15	CREBBP	1	0,86%	3,85%	15	MDM2	2	1,72%	7,69%
16	CTCF	1	0,86%	3,85%	16	MET	2	1,72%	7,69%
17	CTNNB1	1	0,86%	3,85%	17	RAF1	2	1,72%	7,69%
18	CUL3	1	0,86%	3,85%	18	AKT1	1	0,86%	3,85%
19	DNMT3A	10	8,62%	38,46%	19	ALK	1	0,86%	3,85%
20	EGFR	12	10,34%	46,15%	20	APC	1	0,86%	3,85%
21	ERBB2	1	0,86%	3,85%	21	ARAF	1	0,86%	3,85%
22	EZH2	1	0,86%	3,85%	22	ARID1A	1	0,86%	3,85%
23	FGFR1	1	0,86%	3,85%	23	ATM	1	0,86%	3,85%
24	GNAS	1	0,86%	3,85%	24	BARD1	1	0,86%	3,85%
25	HGF	1	0,86%	3,85%	25	BCOR	1	0,86%	3,85%
26	IDH2	1	0,86%	3,85%	26	CDK6	1	0,86%	3,85%
27	JAK2	2	1,72%	7,69%	27	CHEK2	1	0,86%	3,85%
28	KEAP1	2	1,72%	7,69%	28	CREBBP	1	0,86%	3,85%
29	KRAS	3	2,59%	11,54%	29	CTCF	1	0,86%	3,85%
30	MAP2K4	1	0,86%	3,85%	30	CTNNB1	1	0,86%	3,85%
31	MDM2	2	1,72%	7,69%	31	CUL3	1	0,86%	3,85%
32	MET	2	1,72%	7,69%	32	ERBB2	1	0,86%	3,85%
33	MTAP	3	2,59%	11,54%	33	EZH2	1	0,86%	3,85%
34	MYC	1	0,86%	3,85%	34	FGFR1	1	0,86%	3,85%
35	NF1	4	3,45%	15,38%	35	GNAS	1	0,86%	3,85%
36	NF2	1	0,86%	3,85%	36	HGF	1	0,86%	3,85%
37	NRAS	1	0,86%	3,85%	37	IDH2	1	0,86%	3,85%
38	NSD3 (WHSC1L1)	1	0,86%	3,85%	38	MAP2K4	1	0,86%	3,85%
39	PBRM1	1	0,86%	3,85%	39	MYC	1	0,86%	3,85%

40	PDGFRA	1	0,86%	3,85%
41	PIK3CA	1	0,86%	3,85%
42	PRKCI	1	0,86%	3,85%
43	PTEN	1	0,86%	3,85%
44	RAD21	1	0,86%	3,85%
45	RAF1	2	1,72%	7,69%
46	RBM10	1	0,86%	3,85%
47	SMAD4	1	0,86%	3,85%
48	SMARCA4	3	2,59%	11,54%
49	SPOP	1	0,86%	3,85%
50	STAG2	1	0,86%	3,85%
51	STK11	1	0,86%	3,85%
52	TERC	1	0,86%	3,85%
53	TERT	1	0,86%	3,85%
54	TET2	4	3,45%	15,38%
55	TP53	14	12,07%	53,85%
56	XRCC2	1	0,86%	3,85%
57	ZNF703	1	0,86%	3,85%
58	No muts (2 pts)	0	0,00%	0,00%
	Total	116	100,00%	

40	NF2	1	0,86%	3,85%
41	NRAS	1	0,86%	3,85%
42	NSD3 (WHSC1L1)	1	0,86%	3,85%
43	PBRM1	1	0,86%	3,85%
44	PDGFRA	1	0,86%	3,85%
45	PIK3CA	1	0,86%	3,85%
46	PRKCI	1	0,86%	3,85%
47	PTEN	1	0,86%	3,85%
48	RAD21	1	0,86%	3,85%
49	RBM10	1	0,86%	3,85%
50	SMAD4	1	0,86%	3,85%
51	SPOP	1	0,86%	3,85%
52	STAG2	1	0,86%	3,85%
53	STK11	1	0,86%	3,85%
54	TERC	1	0,86%	3,85%
55	TERT	1	0,86%	3,85%
56	XRCC2	1	0,86%	3,85%
57	ZNF703	1	0,86%	3,85%
58	No muts (2 pts)	0	0,00%	0,00%
	Total	116	100,00%	

Total number of patients tested N=26. Total number of genetic abnormalities found n=116. Genes in red may represent clonal hematopoesis.
